# Rad52 mediates class-switch DNA recombination to IgD

**DOI:** 10.1038/s41467-022-28576-2

**Published:** 2022-02-21

**Authors:** Yijiang Xu, Hang Zhou, Ginell Post, Hong Zan, Paolo Casali

**Affiliations:** 1 Department of Microbiology, Immunology & Molecular Genetics, University of Texas Long School of Medicine, UT Health Science Center, San Antonio, TX 78229 USA; 2 Department of Pathology, University of Arkansas School of Medicine, Little Rock, AR 72205 USA; 3 Department of Medicine, University of Texas Long School of Medicine, UT Health Science Center, San Antonio, TX 78229 USA

**Keywords:** DNA recombination, Class switch recombination, Antibodies, B-2 cells

## Abstract

In B cells, IgD is expressed together with IgM through alternative splicing of primary *V*_*H*_*DJ*_*H*_*-Cμ-s-m-Cδ-s-m* RNAs, and also through IgD class switch DNA recombination (CSR) via double-strand DNA breaks (DSB) and synapse of Sμ with σ*δ*. How such DSBs are resolved is still unknown, despite our previous report showing that Rad52 effects the ‘short-range’ microhomology-mediated synapsis of intra-Sμ region DSBs. Here we find that induction of IgD CSR downregulates Zfp318, and promotes Rad52 phosphorylation and recruitment to Sμ and σ*δ*, thereby leading to alternative end-joining (A-EJ)-mediated Sμ-σ*δ* recombination with extensive microhomologies, *V*_*H*_*DJ*_*H*_-C*δs* transcription and sustained IgD secretion. Rad52 ablation in mouse *Rad52*^−/−^ B cells aborts IgD CSR in vitro and in vivo and dampens the specific IgD antibody response to OVA. Rad52 knockdown in human B cells also abrogates IgD CSR. Finally, Rad52 phosphorylation is associated with high levels of IgD CSR and anti-nuclear IgD autoantibodies in patients with systemic lupus erythematosus and in lupus-prone mice. Our findings thus show that Rad52 mediates IgD CSR through microhomology-mediated A-EJ in concert with Zfp318 downregulation.

## Abstract (web summary)

IgD is expressed, predominantly together with IgM, via mRNA alternative splicing, but IgD class switch recombination (IgD CSR) has also been reported. Here the authors show, using Rad52-deficient mouse and human B cells, that IgD CSR is mediated by Rad52 through an alternative, microhomology-based end-joining pathway of DNA repair.

## Introduction

IgD has been an enigmatic antibody class for many years, despite being evolutionarily ancient and highly conserved across species^1–6^. As primordial as IgM, IgD appeared in cartilaginous fishes, amphibians and occurs in fishes, rodents, cattle, and humans^2,7^. As an example, in *Xenopus*, the Igδ exon cluster is in the same position, immediately 3′ of the Igμ locus, as it exists in mammals^7^. In mice and humans, IgD is expressed primarily as a transmembrane IgD receptor together with IgM with identical antigen specificity on naïve mature B cells in the form of BCR. IgD also exists as a secreted antibody. In humans, circulating IgD occurs at concentrations up to more than two-thousand folds greater than IgE (10–250 μg/ml vs. ~0.1 μg/ml), the rarest peripheral blood Ig class. IgD is secreted by IgM^−^IgD^+^ plasmablasts and plasma cells differentiated from B cells in lymphoepithelial organs of aerodigestive mucosae, including palatine and pharyngeal tonsils. IgM^−^IgD^+^ B cells and plasma cells can also be found in the lachrymal, salivary and mammary glands^3^. In addition to existing as a free molecule, IgD can occur on the surface of innate effector cells, including basophils, mast cells, and monocytes^1,8,9^. IgD bound to these cells would enhance immune surveillance and exert proinflammatory and antimicrobial effects^1,8,9^. These include triggering basophils to secret IL-4, IL-5, and IL-13 upon antigen engagement or attenuating basophil or mast cell allergic degranulation induced by IgE co-engagement^1^. Thus, IgD would contribute to mucosal homeostasis by modulating effector cells reactivity to microbial commensals and pathogens^5,6^.

**Fig. 1:**
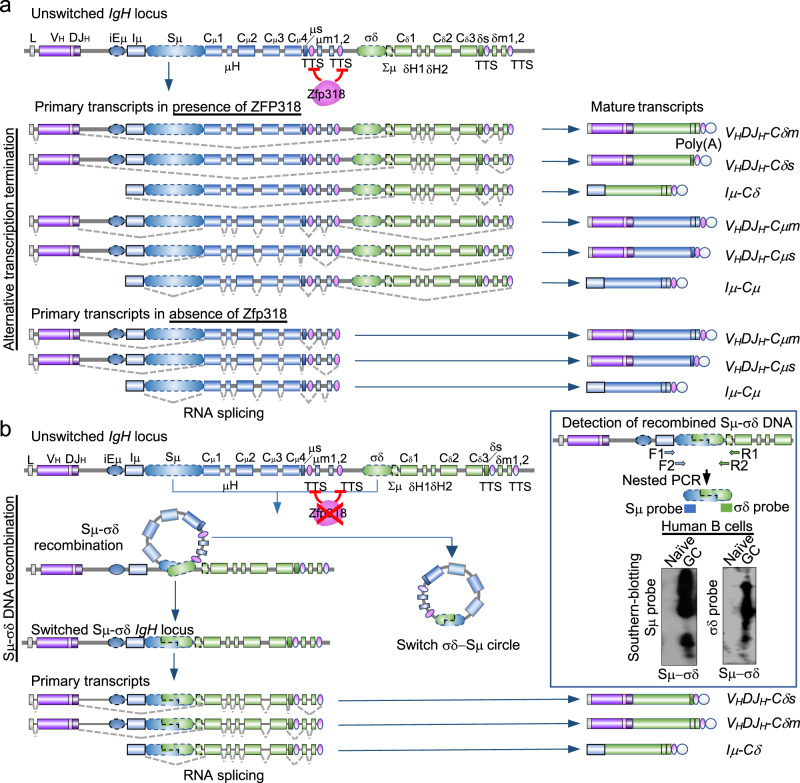
Expression of cell surface and secreted IgD and IgM, as well as *Iμ-Cδ* transcripts by alternative splicing, alternative transcription termination and CSR. **a** Alternative splicing and alternative transcription termination underpin the expression of germline *Iμ-Cμ* and *Iμ-Cδ* transcripts, as well as membrane and secreted IgM and IgD in B cells. Expression of IgD stems from either Zfp318-dependent alternative mRNA splicing or Sμ–σδ CSR. In the presence of Zfp318, which represses the transcription termination sites (TTS) of the Cμ gene, mature B cells constitutively transcribe long primary *V*_*H*_*DJ*_*H*_*-Cμ-Cδs–m* transcripts initiated by the V_H_ promoter. These long primary transcripts undergo alternative splicing which removes intronic regions, leading to dual expression of mature *V*_*H*_*DJ*_*H*_*-Cμs* and *V*_*H*_*DJ*_*H*_*-Cδm* transcripts encoding IgM and IgD. In the absence of Zfp318, transcription stops at Cμ TTS, resulting in a shorter primary transcript, which does not contain Cδ exons, and leads to expression of a mature *V*_*H*_*DJ*_*H*_*-Cμ−s–m* transcript only. Mature B cells also transcribe Iμ*,* Cμ, and Cδ regions under control of the Iμ promoter. When Zfp318 is expressed, unswitched mature B cells constitutively transcribe long primary *Iμ-Cμ−s−m-Cδ–s−m* transcripts, which undergo alternative splicing to removes intronic regions, leading to dual expression of germline *Iμ-Cμ* and *Iμ-Cδ* transcripts. In the absence of Zfp318, *Iμ* promoter-initiated transcription stops at Cμ TTS, and only germline *Iμ-Cμ* transcripts are expressed. **b** Expression of *Iμ-Cδ* transcripts, and membrane and secreted IgD by CSR. Schematic representation of CSR from IgM to IgD. The Sμ region recombines with the σδ region and loops out the intervening DNA, which forms a switch circle. The recombined DNA is transcribed leading to expression of *V*_*H*_*DJ*_*H*_*-Cδ−s–m* and *Iμ-Cδ* transcripts, initiated by the V_H_ and Iμ promoters, respectively—in this case, *Iμ-Cδ* transcripts are generated as post-recombination transcripts. Graphics depict portion of the *IgH* locus and the resulting primary and mature transcripts. Inset depicts the detection of Sμ–σδ junctional DNA (CSR to IgD) by nested PCR amplification followed by Southern-blotting using specific Sμ and σδ probes (Southern-blotting of amplified recombined Sμ–σδ DNA from human naïve and germinal center B cells). The amplified Sμ–σδ DNA is sequenced for further analysis of the junctional sequence as well as identification and census of mutations. iEμ, *IgH* intronic enhancer; Iμ, intervening μ exon; μm, exon encoding the transmembrane region of IgM; δm, exon encoding the secretory piece of IgM; σδ, noncanonical switch-like region 5′ to Cδ; δs, exon encoding the secretory region of IgD; Cδm, exon encoding the transmembrane region of IgD. Dotted gray lines show splicing of primary transcripts to yield secreted and transmembrane forms of IgM and IgD.

Identifying the stimuli and molecular mechanisms that underpin IgD expression is important to understand the regulation of IgD secretion throughout the body. The immediately proximal location of Cμ and Cδ gene loci as an integrated transcriptional unit allows these two Ig isotypes to be coordinately regulated^10,11^. In naive IgM^+^IgD^+^ B cells, (membrane) mIgM and mIgD are co-expressed by alternative splicing of long primary transcripts consisting of rearranged V_H_DJ_H_ exons and downstream Cμ and Cδ exons (*V*_*H*_*DJ*_*H*_*-Cμ-s-m-Cδ-s-m*). Alternative splicing of the same long primary *V*_*H*_*DJ*_*H*_*-Cμ-s-m-Cδ-s-m* transcripts also leads to expression of (secreted) sIgM and sIgD^2,8^. Transcription of long primary *V*_*H*_*DJ*_*H*_*-Cμ-s-m-Cδ-s-m* RNA requires the zinc finger ZFP318 repressor of transcriptional termination, which, as shown with genetically modified mouse models, obliterates the effect of the transcriptional termination sites (TTS) intercalated between the Cμ and Cδ exon clusters^10,11^ (Fig. 1a). IgD can also be expressed through class-switch DNA recombination (CSR), by which IgM^+^IgD^+^ B cells juxtapose *V*_*H*_*DJ*_*H*_ DNA from the Cμ (IgM) to the Cδ (IgD) exons cluster, giving rise to *V*_*H*_*DJ*_*H*_*-Cδm* RNA transcripts and IgM^−^IgD^+^ B cells^1,5,8,9,12^ (Fig. 1b). In human and mouse nasopharyngeal and intestinal lymphoid tissues, a significant proportion of mucosal B cells class-switch to IgM^−^IgD^+^ B cells, which subsequentially differentiate to plasmablasts and plasma cells^1,3,5,6^. Generally, CSR to IgD (Cδ) is a less frequent event than CSR to IgG (Cγ), IgA (Cα) or IgE (Cε), perhaps a reflection among other factors of the peculiar structure of the pseudo-switch σδ region lying immediately upstream of Cδ exons. Compared to the canonical Sμ, Sγ, Sα and Sε regions lying 5′ of the respective *Igμ*, *Igγ*, *Igα*, and *Igε* loci, σδ is shorter and contain differing motifs of nucleotide (nt) repeats^2,5,8,13,14^. These would provide an unconventional substrate for AID-mediated insertion of DSBs, possibly more prone to end-resection and generation of single-strand overhangs for Sμ–σδ recombination and expression of post-recombination *V*_*H*_*DJ*_*H*_*-Cδ* RNA transcripts^2,8,13–15^.

Unlike CSR to IgG, IgA, and IgE, the mechanism of CSR to IgD remains unknown. Recombination involving Sμ DSB ends with DSB ends in downstream Sμ, Sγ, Sα, or Sε region is effected by non-homologous end-joining (NHEJ), one of the two major DNA DSB repair pathways, the other being homologous recombination (HR)^16,17^. HR accurately repairs resected (staggered) DSB ends using a sister chromatid as a homologous single-strand template during cell cycle S-G2. It critically effects error-free DSB repair in somatic cells and helps orchestrate chromosome segregation in meiosis. In contrast to HR, NHEJ is a homology-independent error-prone DSB repair process. It synapses blunt or virtually blunt DSB ends that lack substantial joining complementarity to form “direct” junctions, predominantly in G1 but also throughout the whole cell cycle^16^. NHEJ requires Ku70/Ku86 and in CSR mediates efficient long-range synapses of Sμ DSB ends with Sγ, Sα, and Sε DSB ends, leading to IgG, IgA, and IgE^15^. The finding, however, that reduction or deletion of Ku70/Ku86 led to reduced but still substantial CSR to IgG1 and IgG3 supported the existence of an alternative CSR end-joining (A-EJ) pathway^18–20^. This, like HR, would join resected DSB ends, thereby giving rise to S–S junctions involving microhomologies. Unlike HR, however, the A-EJ pathway juxtaposes DSB overhangs to be joined without using a homologous template as a guide. Rather, it utilizes differing extents of sequence complementarity (homology) between the upstream and downstream resected DSB overhangs to align the to-be DNA junctions^21^. As we have shown, HR factor Rad52 competes with NHEJ factors Ku70/Ku86 for binding to S region DSB ends and synapses DSB ends by A-EJ through microhomology-mediated end-joining (MMEJ)^20^, as inferred from increased NHEJ-mediated IgG, IgA, and IgE CSR events with even fewer S–S junction microhomologies in *Rad52*^*−/−*^ B cells in vivo and in vitro^20^. This together with the increased CSR to IgD in B cells lacking 53BP1, which protects S regions DSB ends from resection and facilitates long-range NHEJ to IgG, IgA, and IgE^22–24^, as well as other findings of ours showing reduced intra-Sμ region DSB short-range rejoining in *Rad52*^*−/−*^ B cells^20^ led us to hypothesize that Rad52 mediates CSR to IgD through short-range Sμ–σδ DSB recombination by annealing to Sμ and σδ DSB single-strand resected ends.

By activating *Rad52*^*−/−*^ mouse B cells and *RAD52* siRNA knockdown human B cells with different stimuli together with molecular genetic methods, here we show that Rad52 critically effects Sμ–σδ DNA recombination and CSR to IgD, through a microhomology-mediated (MM) A-EJ process which is associated with junctional Sμ–σδ somatic point-mutations. For this synaptic process, Rad52 is phosphorylated and recruited to Sμ and σδ regions, concomitant with downregulation of the TTS repressor Zfp318. Rad52-mediated Sμ–σδ CSR is required to mount an IgD-specific antibody response and is upregulated in systemic lupus erythematosus (SLE) patients and lupus mice, leading to high levels of total IgD and antinuclear antigen IgD autoantibodies. Thus, our experiments unveil a previously unrecognized and essential role of the Rad52 HR factor in a mammalian MM A-EJ DSB repair process which underpins a unique modality of CSR in health and disease.

## Results

### Definition of stimuli that induce Sμ–σδ CSR in mouse and human B cells

Toward testing our hypothesis that Rad52 mediates CSR to IgD, we first identified the stimuli that induce naïve IgM^+^IgD^+^ B cells to undergo Sμ–σδ recombination. In naïve IgM^+^IgD^+^ B cells, mIgM and mIgD are expressed by alternative splicing of long primary *V*_*H*_*DJ*_*H*_*-Cμ-s-m-Cδ-s-m* mRNAs—the Cδ locus is located immediately downstream of the Cμ locus in the same transcriptional unit, allowing these two loci to be coordinately regulated at the transcriptional level^1,2,4,6^ (Fig. 1a). As CSR can be induced in a T-dependent or T-independent fashion^15,25^, we used CD40 ligand CD154 (for mouse and human B cells), TLR4 ligand LPS (for mouse B cells), and TLR9 ligand CpG (for human B cells) in conjunction with different cytokines and/or BCR-cross-linking to induced CSR to IgD. Unlike IgG, IgA, and IgE, IgD can be expressed in the absence of CSR on surface of mature naive IgM^+^IgD^+^ B cells, at high levels in a Zfp318-dependent fashion^10,11^. Accordingly, *Aicda* knockout B cells, in which CSR to IgD is abolished, can display robust surface IgD, at a level comparable to wild-type B cells^5^. Therefore, expression of IgD, as detected by surface/intracellular staining and/or flow cytometry, does not necessarily reflect Sμ–σδ recombination events, thereby making detection of recombined Sμ–σδ DNA the direct and positive proof of CSR to IgD.

**Fig. 2:**
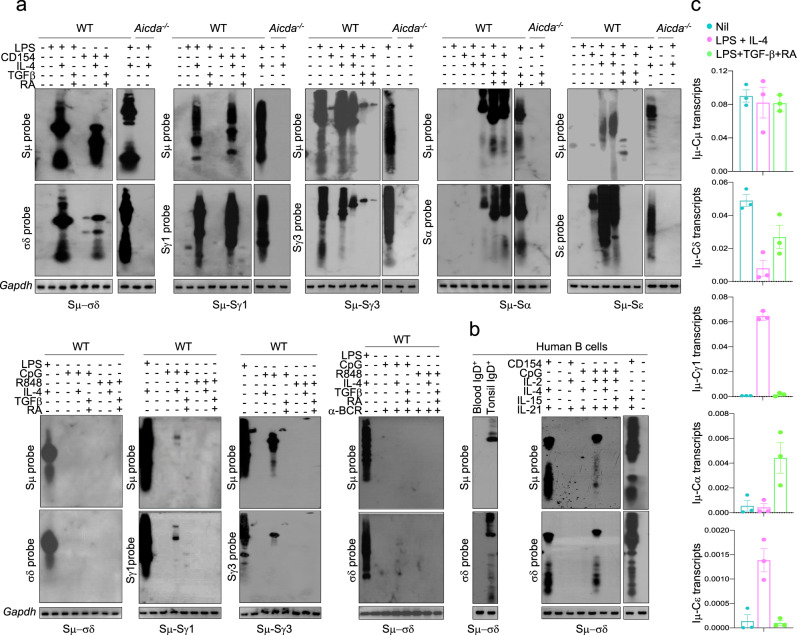
Identification of stimuli inducing CSR to IgD and Sμ–σδ junctions in mouse and human B cells. **a** Unstimulated mouse (wildtype) C57BL/6 B cells and naïve *Aicda*^*−/−*^ B cells (nil), or C57BL/6 and naïve *Aicda*^*−/−*^ B cells stimulated for 96 h with LPS, LPS plus IL-4, LPS plus TGF-β and RA, CD154, CD154 plus IL-4, CD154 plus TGF-β and RA, CpG alone, CpG plus IL-4, CpG plus TGF-β and RA, R848 alone, R848 plus IL-4, R848 plus TGF-β and RA, or CpG plus IL-4, or R848 plus IL-4 in the presence of anti-BCR Ab were analyzed for recombined Sμ–σδ, as well as recombined Sμ–Sγ1, Sμ–Sγ3, Sμ–Sα, and Sμ–Sα DNA by nested PCR using forward Iμ and reverse Cδ primers, or forward Iμ and reverse Sγ1, Sγ3, Sα or Sε primers, respectively, followed by Southern-blotting using a specific Sμ, σδ, Sγ1, Sγ3, Sα, or Sε probe, as indicated. **b** Recombined Sμ–σδ DNA in human tonsil IgD^+ ^B cells, blood naive IgM^+^IgD^+^ B cells, or blood naive IgM^+^IgD^+^ B cells stimulated with CD145 or CpG plus IL-2 and IL-21, IL-4 and IL-21, or IL-2, IL-4 and IL-21, or IL-15 and Il-21, were analyzed 120 h post-stimulation by nested PCR using forward Iμ and reverse Cδ primers, followed by Southern-blotting using specific human Sμ or σδ probe. Data are one representative of three independent experiments yielding comparable results. **c** Quantification of germline/post-recombination Iμ-Cδ transcripts, post-recombination *Iμ-Cγ1*, *Iμ-Cα*, and *Iμ-Cε* transcripts in wildtype C57BL/6 B cells stimulated with nil, LPS plus IL-4, or LPS plus TGF-β and RA, analyzed 72 h post-stimulation by qRT-PCR and normalized to *β-Actin* expression. Each dot represents data obtained with B cells from an individual mouse (*n* = 3 per group). Data are mean ± SEM. Source data are provided as a Source Data file.

We detected recombined Sμ–σδ, Sμ–Sγ1, Sμ–Sγ3, Sμ–Sα, and Sμ–Sε DNAs by specific nested PCRs followed by positive identification of amplified DNA by blotting and hybridization with specific internal DNA probes (Fig. 1 inset), complemented by sequencing of junctional Sμ–σδ or Sμ–S_X_ DNAs. Of all stimuli used, only LPS or CD154 plus IL-4 induced CSR to IgD in mouse B cells (Fig. 2a), and only CpG plus IL-2 and IL-21, or CD154 plus IL-4 or IL-15 and IL-21 induced CSR to IgD in human B cells (Fig. 2b). CSR to IgD was also detected in vivo in tonsil B cells. The effectiveness of the stimuli that did not induce CSR to IgD was verified by the respective induction of expected Sμ–Sγ1, Sμ–Sγ3, Sμ–Sα or Sμ–Sε DNA recombination (IgG1, IgG3, IgA or IgE) (Fig. 2a)—no CSR to IgD, IgG, IgA or IgE occurred in *Aicda*^−/−^ B cells. In all cases, CSR was further confirmed by detection of post-recombination *Iμ-Cγ1, Iμ-Cγ3, Iμ-Cα* and *Iμ-Cε* transcripts at 72 h of culture—as post-recombination *Iμ-Cδ* transcripts are indistinguishable from germline *Iμ-Cδs-m* RNA transcripts and consistent with high levels of the latter in naïve B cells, *Iμ-Cδ* amplification products were less abundant in class-switched IgD than naïve B cells (Figs. 1, 2c). Thus, only select stimuli induce CSR to IgD in mouse and human B cells.

### Sμ–σδ junctions are enriched in microhomologies and abetted by somatic mutations in mouse and human B cells

**Fig. 3:**
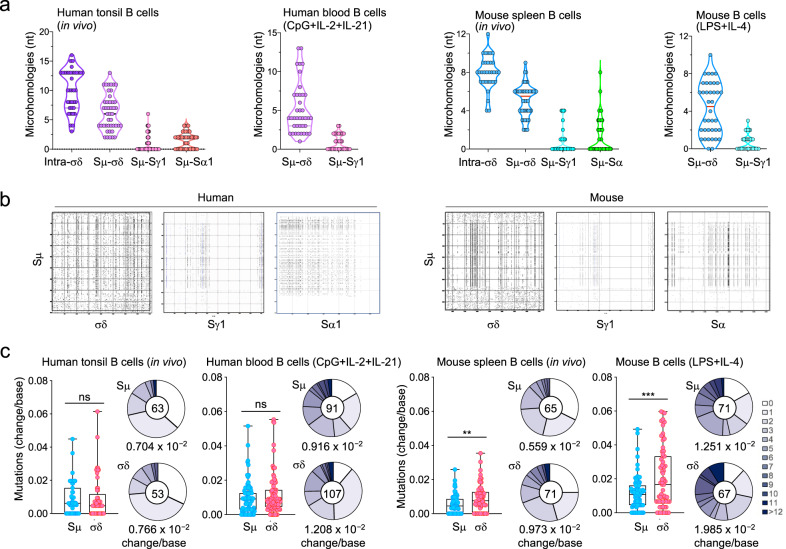
Mouse and human Sμ–σδ DNA recombination junctions contain microhomologies and somatic mutations. **a** Amplified DNAs from junctional intra-σδ deletions as well as Sμ–σδ, Sμ–Sγ1 and Sμ–Sα1 recombinations from human tonsil B cells or human peripheral blood naïve IgM^+^IgD^+^ B cells stimulated with CpG plus IL-2 and IL-21 and cultured for 120 h, OVA-immunized C57BL/6 mouse spleen B cells or C57BL/6 mouse naïve IgM^+^IgD^+^ B cells stimulated with LPS plus IL-4 and cultured for 96 h were amplified and sequenced by MiSeq. The length and numbers of nucleotide overlaps (microhomologies) in intra-σδ deletions, Sμ–σδ, Sμ–Sγ1, and Sμ–Sα1 junctional DNAs are shown by violin plots. Each dot represents a unique junctional sequence (*n* = 45 per group). **b** Human and mouse Sμ and σδ regions consist of repetitive motifs, which are better-suited substrates for Rad52-mediated MMEJ than those in Sμ and Sγ1 or Sμ and Sα. As such, they can facilitate the formation of microhomologies. Repetitive sequence elements in mouse and human Sμ, σδ, Sγ1 and Sα that can potentially form microhomologies were identified by Pustell Matrix dot plot using MacVector software and are depicted by small dots. Intensity of dots depicts frequency and degree of complementarity of respective sequences. **c** Somatic point-mutations in Sμ and σδ regions abetting recombined Sμ−σδ DNA junctions in IgD class-switched human and mouse B cells in vivo and in vitro. Mutations were identified in a 48–506 nt stretch of Sμ or σδ regions in unique Sμ–σδ DNA recombination sequences. Each dot represents an individual sequence. Sequence data were pooled from three individuals in each group. Box and whiskers plots show median, quartiles, maximum and minimum of mutation frequencies in Sμ and σδ regions. In pie charts, the size of slices denotes the proportion of transcripts with the same number of mutations and the gray hue denotes the number of point mutations per transcript. Center of pie shows the total number of independent sequences analyzed. Below the pie charts is the overall mutation frequency (change/base). **p < 0.01, ***p < 0.001, ns: not significant (unpaired two-tailed *t-*test). Source data are provided as a Source Data file.

The mechanisms effecting CSR can leave an S–S synaptic signature^20,26^. As we previously showed, Rad52 mediates A-EJ of resected DSB ends by juxtaposing overhangs with nucleotide complementarities, thereby giving rise to Sμ-Sx DNA junctions with microhomologies^20^. Next-generation sequencing of >100,000 recombined Sμ-Sx DNA junctions from mouse and human B cells in vitro and in vivo showed that Sμ–σδ junctions contained significantly more microhomologies (*p* < 0.01) than Sμ–Sγ1 or Sμ–Sα DNA junctions (representative frequencies and lengths of microhomologies in human and mouse B cells are depicted in Fig. 3a; representative human and mouse intra-σδ and junctional Sμ-Sx sequences are depicted in Supplementary Figs. 1–3), indicating that an MMEJ^21^ process underpinned Sμ–σδ synapses. In both human and mouse B cells, the microhomologies in Sμ–σδ junctions were significantly more extensive than those in Sμ–Sγ1 and, to a lesser extent, Sμ–Sα junctions (Supplementary Figs. 1–4). As one example, in human tonsil B cells, 100% of analyzed Sμ–σδ junctions contained microhomologies, consisting of 2–13 nucleotides (mean = 6.30), while only 21% of Sμ–Sγ1 junctions contained microhomologies, consisting of 1–6 nucleotides (mean = 0.72) (Fig. 3a). Interestingly, there were a few common S–S sequences shared by recombined Sμ–σδ DNA junctions in human tonsil B cells and blood naïve B cells stimulated in vitro by CpG plus IL-2 and IL-21, suggesting that select Sμ and σδ DSB hotspots underpin Sμ–σδ DNA recombinations. A high frequency of microhomologies was also evident in the synaptic repair process of intra-σδ DSBs, evocative of what we showed in intra-Sμ DSBs^20^. Consistent with the greatest occurrence of microhomologies in Sμ–σδ junctions, Sμ is better suited for complementary DNA single-strand annealing with σδ than Sγ1 or, to a lesser extent, Sα (mouse) or Sα1 (human), based on various numbers and contexts of these DNA regions discrete motifs, such as [G_n_]AGCT repeats (Sμ, Sγ, and Sα) or AGCTGAGCTG repeats (Sμ and σδ), as revealed by Pustell Matrix dot-plot analysis (MacVector software) (Fig. 3b). During CSR, somatic mutations are introduced into the (upstream) donor Sμ and the (downstream) acceptor Sx regions, as effected by AID-targeting of these DNA regions. Indeed, Sμ–σδ DNA junctions were associated with somatic point-mutations in human and mouse B cells. These were more frequent in the σδ region than the Sμ area abetting the Sμ–σδ junction (e.g., 0.559 × 10^−2^ vs. 0.973 × 10^*−*2^ change/base in mouse spleen B cells in vivo and 1.251 × 10^−2^ vs. 1.985 × 10^*−*2^ change/base in mouse B cells stimulated by LPS plus IL-4 in vitro) (Fig. 3c). Thus, the high frequency of microhomologies in Sμ–σδ junctions supports a role of Rad52 in mediating CSR to IgD, while the somatic point-mutations in σδ reflects the targeting by AID.

### Rad52 is critically required for Sμ–σδ synapsing

**Fig. 4:**
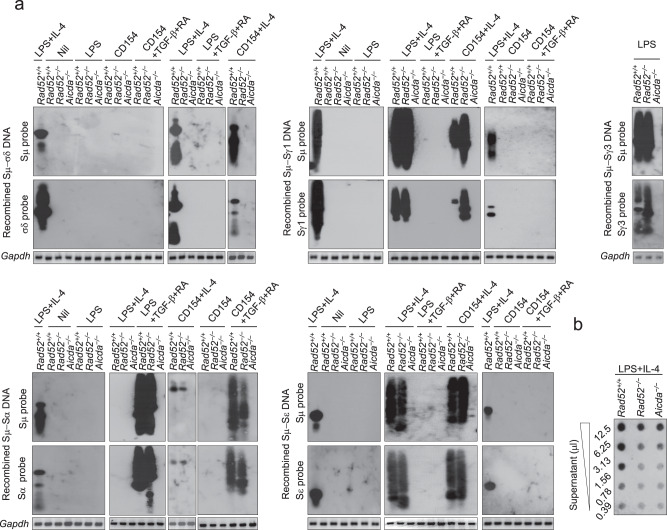
Rad52 mediates Sμ–σδ DNA recombination leading to IgD secretion. **a** Recombined Sμ–σδ, Sμ−Sγ1, Sμ−Sα, and Sμ−Sε DNAs in mouse *Rad52*^*+/+*^*, Rad52*^*−/−*^ and *Aicda*^*−/−*^ naïve IgM^+^IgD^+^ B cells stimulated with nil, LPS alone, LPS plus IL-4, LPS plus TGF-β and RA, CD154 alone, CD154 plus IL-4, or CD154 plus TGF-β and RA, as well as Sμ–Sγ3 in *Rad52*^*+/+*^*, Rad52*^*−/−*^ and *Aicda*^*−/−*^ B cells stimulated with LPS only, were analyzed 96 h post-stimulation by specific nested PCR using forward Iμ and reverse Cδ, Sγ1, Sγ3, Sα, or Sε primers, respectively, followed by Southern-blotting using specific Sμ, σδ, Sγ1, Sγ3, Sα, or Sε probe, as indicated. Data are one representative of three independent experiments yielding comparable results. **b** IgD titers in culture (96 h) fluid of *Rad52*^*+/+*^, *Rad52*^*−/−*^ or *Aicda*^*−/−*^ B cells stimulated with LPS plus IL-4, as measured by dot-blotting (two-fold serial diluted culture fluid) using a rat anti-mouse IgD mAb. Data are one representative of five independent experiments yielding comparable results. Source data are provided as a Source Data file.

Having established that LPS or CD154 pus IL-4 induced CSR to IgD in mouse B cells, we used these stimuli and *Rad52*^*−/−*^ B cells together with appropriate controls (LPS alone, LPS plus TGF-β and RA, CD154 alone, or CD154 plus TGF-β and RA) and the same approach used in the experiments of Fig. 2 to investigate whether Rad52 was required for CSR to IgD. LPS plus IL-4 and CD154 plus IL-4 failed to induce Sμ–σδ recombination in *Rad52*^*−/−*^ B cells but not in *Rad52*^*+/+*^ B cells, while either treatment efficiently induced Sμ–Sγ1 and Sμ–Sε recombinations in the same *Rad52*^*−/−*^ B cells (Fig. 4a)—in *Rad52*^*−/−*^ B cells, LPS, and LPS or CD154 plus TGF-β and RA induced CSR to IgG3 and IgA, respectively. As expected, CSR to IgD as well as IgG, IgA, and IgE was ablated in *Aicda*^*−/−*^ B cells. Finally, the failure of *Rad52*^*−/−*^ B cells and *Aicda*^*−/−*^ B cells to undergo CSR to IgD was associated with significantly decreased secretion of IgD (Fig. 4b). Thus, Rad52 is critical for Sμ–σδ DNA recombination and seemingly important for IgD secretion.

### Rad52 is required to mount an antigen-specific IgD antibody response

**Fig. 5:**
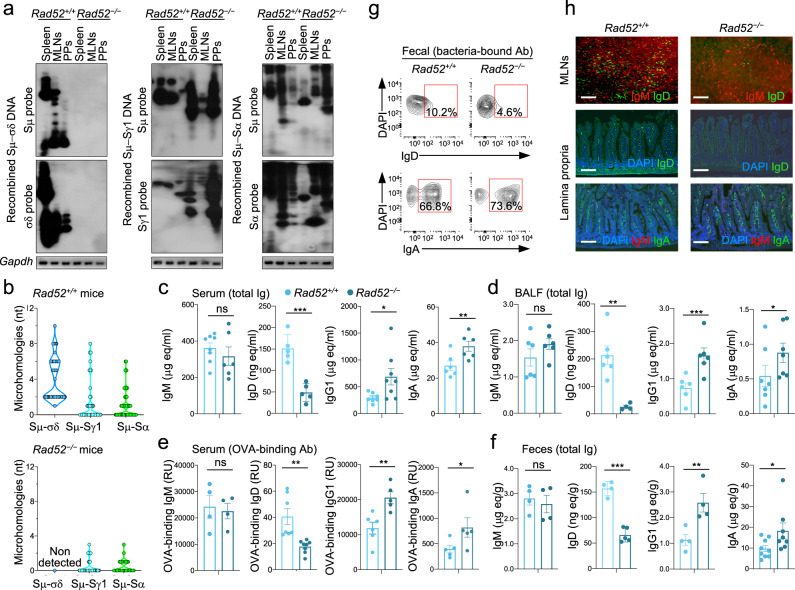
*Rad52* deletion ablates in vivo Sμ–σδ DNA recombination and reduces IgD production. *Rad52*^+/+^ and *Rad52*^*−/−*^ mice were immunized with OVA in alum i.p. **a** Recombined Sμ–σδ, Sμ−Sγ1, and Sμ−Sα DNAs in spleen, mesenteric lymph nodes (MLNs), and Peyer’s patches (PPs) B cells, as analyzed by nested PCR using forward Iμ and reverse Cδ, Sγ1, or Sα primers, respectively, followed by Southern-blotting using specific Sμ, σδ, Sγ1, or Sα probe, as indicated. Data are one representative of three independent experiments yielding comparable results. **b** Sμ–σδ, Sμ–Sγ1, and Sμ–Sα junctional DNAs were amplified by nested PCR and sequenced by MiSeq. The length and numbers of nucleotide overlaps (microhomologies) in Sμ–σδ, Sμ−Sγ1, and Sμ−Sα junctional DNAs are shown by violin plots. Each symbol represents a unique sequence (*n* = 45 per group). **c**–**f** Titers of total IgD in serum, BALF, and feces, as analyzed by dot-blotting using rat anti-mouse IgD mAb—titers of total IgM, IgD, IgG1, and IgA as well as OVA-binding IgM, IgD, IgG1, and IgA as analyzed by specific ELISAs. Each dot represents datum from one individual mouse (*n* = 5–8 per group, as indicated). Data are mean ± SEM. *p < 0.05, **p < 0.01, ***p < 0.001, ns: not significant (unpaired two-tailed *t*-test). No adjustments were made for multiple comparisons. **g** Bacteria-bound IgD and IgA in feces as analyzed by flow cytometry. **h** IgM, IgD, and IgA positive cells in MLNs and lamina propria as visualized by fluorescence microscopy. Scale bar = 100 μm. Data in **g**, **h** are representative of three independent experiments. Source data are provided as a Source Data file.

To determine the role of Rad52 in mediating a specific IgD antibody response, we immunized *Rad52*^*−/−*^ and *Rad52*^*+/+*^ mice with ovalbumin (OVA, 20 μg i.p., three times). *Rad52*^*−/−*^ mice showed no Sμ–σδ recombination in spleen, mesenteric lymph nodes (MLNs), or Peyer’s patch B cells (Fig. 5a). The lack of CSR to IgD was specific, as B cells in such mice showed Sμ–Sγ1 and Sμ–Sα DNA recombinations like mouse *Rad52*^*+/+*^ B cells, which also underwent Sμ–σδ recombination. In *Rad52*^*−/−*^ mice, B cells showed Sμ–Sγ1 and Sμ–Sα DNA junctions with fewer and shorter microhomologies than in *Rad52*^*+/+*^ mice (Fig. 5b and Supplementary Figs. 5,6), a reflection of the lack of Rad52^20^. *Rad52*^*−/−*^ mice also showed significantly decreased total and OVA-specific IgD in circulating blood, bronchoalveolar lavage (BALF), feces (free or bound to fecal bacteria), and IgD-producing cells in MLNs and lamina propria, as compared to their *Rad52*^*+/+*^ mouse counterparts (Fig. 5c–h and Supplementary Fig. 7a). This contrasted with the normal or elevated total and OVA-specific IgM, IgG1, and IgA levels in the same *Rad52*^*−/−*^ mice, as predicted based on our previous findings^27^. Thus, Rad52 is critical for mounting an antigen-specific class-switched IgD response.

### Rad52 is modulated and phosphorylated by IgD CSR-inducing stimuli, and it is recruited to Sμ and σδ

**Fig. 6:**
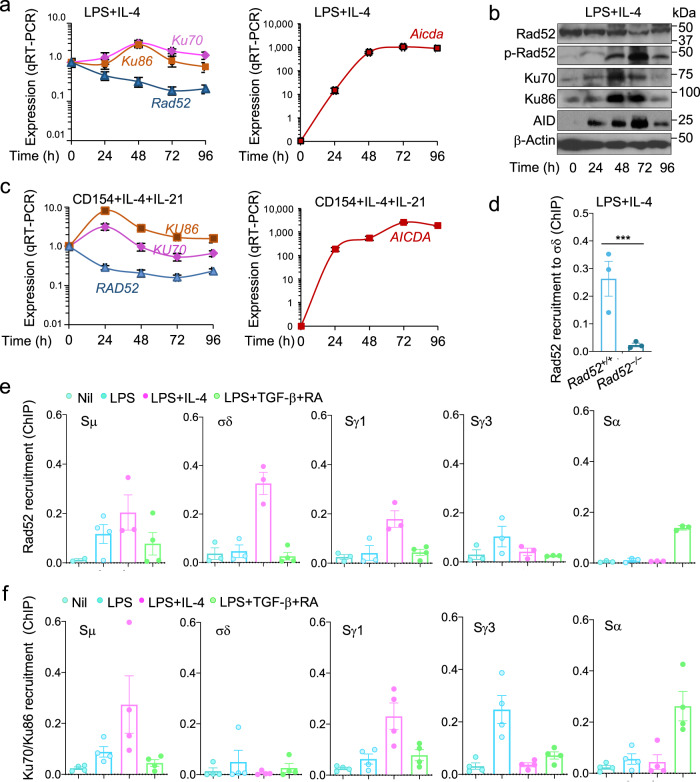
Rad52 is phosphorylated and recruited to Sμ and σδ in B cells induced to undergo IgD CSR. **a** C57BL/6 mouse naïve IgM^+^IgD^+^ B cells were stimulated with LPS plus IL-4 and cultured for 0, 24, 48, 72 and 96 h. *Rad52*, *Ku70*, *Ku86*, and *Aicda* transcripts were analyzed by real-time qRT-PCR, normalized to *β-Actin* expression, and depicted as relative to the expression in unstimulated B cells (set as 1.0). Data are mean ± SEM of three independent experiments. **b** Expression of Rad52, phosphorylated Rad52 (p-Rad52), AID, Ku70, Ku86, and β-Actin proteins in mouse B cells stimulated with LPS plus IL-4 (as in **a**), as analyzed by specific immunoblotting. Data are one representative of three independent experiments yielding comparable results. **c** Human peripheral blood naive IgM^+^IgD^+^ B cells were stimulated with CD154 plus IL-4 and IL-21 and cultured for 0, 24, 48, 72 and 96 h. *RAD52*, *KU70*, *KU86*, and *AICDA* transcripts were analyzed by real-time qRT-PCR, normalized to *β-ACTIN* expression, and depicted as relative to the expression in unstimulated B cells (set as 1.0). Data are mean ± SEM of three independent experiments. **d** Recruitment of Rad52 to σδ region DNA, as analyzed by ChIP-qPCR assays in mouse *Rad52*^*+/+*^ and *Rad52*^*−/−*^ B cells stimulated with LPS plus IL-4 and cultured for 72 h. Data are expressed as percent of pre-IP input for each sample (mean ± SEM of three independent experiments). ***p < 0.001 (unpaired two-tailed *t*-test). **e**, **f** C57BL/6 mouse naïve IgM^+^IgD^+^ B cells were stimulated with nil, LPS alone, LPS plus IL-4, or LPS plus TGF-β and RA and cultured for 72 h. Recruitment of Rad52 (**e**) and Ku70/Ku86 (**f**) to Sμ, σδ, Sγ1, Sγ3, and Sα region DNA, as analyzed by ChIP-qPCR assays. Data are mean ± SEM of three or four independent experiments. Source data are provided as a Source Data file.

We analyzed *Rad52, Ku70, Ku86*, and *Aicda* transcripts as well as the respective Rad52, Ku70, Ku86, and AID proteins, including phosphorylated Rad52 (p-Rad52 has been shown to display enhanced single-strand DNA annealing activity^28^), in B cells induced to undergo CSR to IgD. Mouse naïve IgM^+^IgD^+^ B cells stimulated by LPS plus IL-4 and human naïve IgM^+^IgD^+^ B cells stimulated by CD154 plus IL-4 and IL-21 increased *Ku70/Ku86* and Ku70/Ku86 expression at 24–48 h concomitant with significantly greater expression of *Aicda* and AID, which was virtually undetectable at time 0, while somewhat downregulating *Rad52* and Rad52. Rad52 protein, however, was increasingly phosphorylated within the same time range (Fig. 6a–c). Further supporting its role in CSR to IgD, Rad52 was recruited to Sμ, σδ (and Sγ1) in B cells stimulated by LPS plus IL-4, which induced Sμ–σδ (and Sμ–Sγ1) DNA recombination, but not by stimuli that did not induce Sμ–σδ recombination, i.e., LPS alone or LPS plus TGF-β and RA, as shown by chromatin immunoprecipitation (ChIP) using an anti-Rad52 Ab—the specificity of the ChIP Rad52 recruitment assay being emphasized by the lack of chromatin immunoprecipitation in *Rad52*^*−/−*^ B cells (Fig. 6d, e). Recruitment of Rad52 but not Ku70/Ku86 to σδ in CSR to IgD, as induced by LPS plus IL-4, contrasted with that of Ku70/Ku86 to Sγ3 and Sα regions as induced in CSR to IgG3 and IgA (Fig. 6f), possibly a reflection of the competition of these HR and NHEJ elements for binding to S region DSB ends^20^. Notably, LPS plus IL-4 induced recruitment of Rad52 but not Ku70/Ku86 to σδ, while inducing mostly Ku70/Ku86 recruitment to Cγ1, consistent with the efficient LPS pus IL-4 induction of CSR to IgG1, mediated mainly by NHEJ^20^. Thus, Rad52 modulation and, importantly, Rad52 phosphorylation are induced by IgD CSR-inducing stimuli to recruit Rad52 to σδ region DNA.

### Stimuli that induce Sμ–σδ DNA recombination downregulate ZFP318/Zfp318 and lead to IgD secretion

**Fig. 7:**
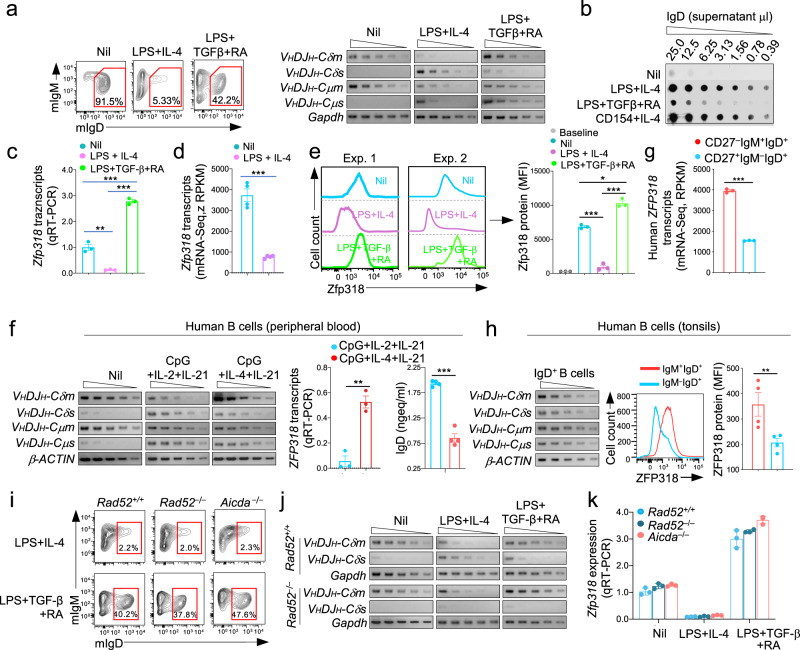
Stimuli inducing Sμ–σδ DNA recombination downregulate ZFP318/Zfp318 in human and mouse B cells. **a** C57BL/6 mouse naïve IgM^+^IgD^+^ B cells were stimulated with nil, LPS plus IL-4 or LPS plus TGF-β and RA. Surface expression of IgM and IgD were analyzed 96 h post-stimulation by flow cytometry. Expression of *V*_*H*_*DJ*_*H*_*−Cδm*, *V*_*H*_*DJ*_*H*_*−Cδs*, *V*_*H*_*DJ*_*H*_*−Cμm*, and *V*_*H*_*DJ*_*H*_*−Cμs* transcripts were analyzed 72 h post-stimulation by semi-quantitative RT-PCR using serial two-fold dilution of cDNA templates. Data are representative of three independent experiments. **b** IgD in supernatant from cultures (96 h) of C57BL/6 naïve IgM^+^IgD^+^ B cell stimulated with nil, LPS plus IL-4, LPS plus TGF-β and RA, or CD154 plus IL-4, as analyzed by dot-blotting using rat anti-mouse IgD mAb. Data are representative of 5 independent experiments. **c** Expression of *Zfp318* transcripts in mouse naïve B cells stimulated with nil, LPS plus IL-4, or LPS plus TGF-β and RA, as analyzed 72 h post-stimulation by qRT-PCR and normalized to *β-Actin* expression and depicted relative to the average expression in unstimulated B cells (set as 1). Data are mean ± SEM of three independent experiments. **p < 0.01, ***p < 0.001 (unpaired two-tailed *t*-test). **d** Expression of *Zfp318* transcripts in unstimulated mouse naïve B cells (Nil) and mouse naïve B cells stimulated with LPS plus IL-4 for 72 h, as analyzed by mRNA-Seq. Data are mean ± SEM of four independent experiments. ***p < 0.001 (unpaired two-tailed *t*-test). **e** Zfp318 protein level in mouse naïve B cells stimulated with nil, LPS plus IL-4, or LPS plus TGF-β and RA, as analyzed 96 h post-stimulation by intracellular staining with rabbit anti-Zfp318 Ab in flow cytometry. Bars in the right panel represent level of MFI (mean ± SEM) from three independent experiments. *p < 0.05, ***p < 0.001 (unpaired two-tailed *t*-test). **f** Human blood naïve IgM^+^IgD^+^ B cells were stimulated with nil, CpG plus IL-2 and IL-21 or CpG plus IL-4 and IL-21; *V*_*H*_*DJ*_*H*_*−Cδm*, *V*_*H*_*DJ*_*H*_*−Cδs, V*_*H*_*DJ*_*H*_*−Cμm*, and *V*_*H*_*DJ*_*H*_*−Cμs* transcript levels were measured 72 h post-stimulation by semi-quantitative RT-PCR with serial two-fold dilution of cDNA templates—data are representative of three independent experiments (left panels). Expression of *ZFP318* transcripts as analyzed 72 h post-stimulation by qRT-PCR and normalized to *HPRT* expression (**f** middle panel)—data are mean ± SEM of three independent experiments. Secreted IgD in supernatants of the human B cell cultures, as analyzed 120 h post-stimulation by specific ELISA (**f** right panel)—data are mean ± SEM of four independent experiments. **p < 0.01, ***p < 0.001 (unpaired two-tailed *t*-test). **g** Expression of *ZFP318* transcripts in human naïve CD27^−^IgM^+^IgD^+^ B cells and memory CD27^+^IgM^−^IgD^+^ B cells isolated from peripheral blood of healthy subjects, as analyzed by mRNA-Seq. Data are mean ± SEM of three independent experiments. **h** Expression of *V*_*H*_*DJ*_*H*_*−Cδm, V*_*H*_*DJ*_*H*_*−Cδs, V*_*H*_*DJ*_*H*_*−Cμm*, and *V*_*H*_*DJ*_*H*_*−Cμs* transcripts in human tonsil IgD^+^ B cells, as analyzed by semi-quantitative RT-PCR involving serial two-fold dilution of cDNA templates (left panel)—data are representative of three independent experiments. Expression of ZFP318 protein in human tonsil IgM^+^IgD^+^ B cells and IgM^−^IgD^+^ B cells, as analyzed by intracellular staining with anti-Zfp318 Ab in flow cytometry (middle panel)—data are representative of four independent experiments (mean ± SEM, right panel). **p < 0.01, ns: not significant (unpaired two-tailed *t*-test). **i** Surface expression of IgM and IgD in mouse naïve *Rad52*^*+/+*^*, Rad52*^*−/−*^ and *Aicda*^*−/−*^ B cells stimulated with LPS plus IL-4, or LPS plus TGF-β and RA, as analyzed 96 h post-stimulation by flow cytometry. Data are representative of three independent experiments. **j** Expression of *V*_*H*_*DJ*_*H*_*−Cδm* and *V*_*H*_*DJ*_*H*_*−Cδs* transcripts in mouse naïve *Rad52*^*+/+*^ and *Rad52*^*−/−*^ B cells stimulated with nil, LPS plus IL-4 or LPS plus TGF-β and RA, as analyzed 72 h post-stimulation by semi-quantitative RT-PCR using serial two-fold dilution of cDNA templates. Data are representative of three independent experiments. **k** Rad52 or AID deficiency does not alter *Zfp318* expression. Expression of *Zfp318* transcripts in mouse naïve *Rad52*^*+/+*^*, Rad52*^*−/−*^ and *Aicda*^*−/−*^ B cells stimulated with nil, LPS plus IL-4, or LPS plus TGF-β and RA, as analyzed 72 h post-stimulation by qRT-PCR and normalized to *β-Actin* expression, as depicted relative to expression in unstimulated B cells (set as 1). Data are mean ± SEM of three independent experiments. No adjustments were made for multiple comparisons. Source data are provided as a Source Data file.

**Fig. 8:**
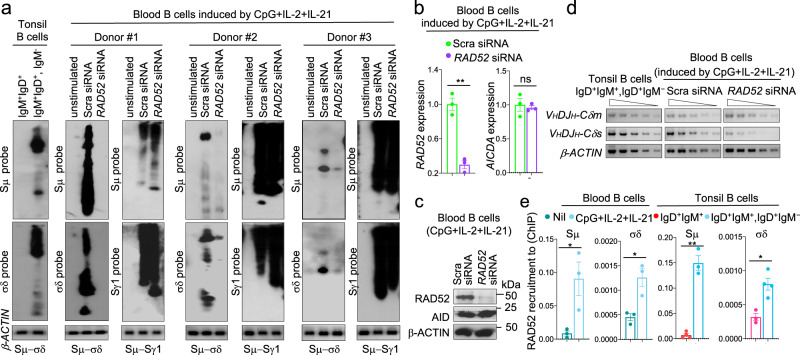
RAD52 is required for Sμ–σδ DNA recombination in human B cells. **a** Human blood naïve IgM^+^IgD^+^ B cells were transfected with specific *RAD52* siRNA or scrambled (Scra) siRNA and stimulated by CpG plus IL-2 and IL-21. Recombined Sμ–σδ and Sμ–Sγ1 DNA in the transfected B cells 120 h after *RAD52* siRNA transfection, as well as Sμ–σδ DNA in tonsil IgM^*–*^IgD^+^ and blood naive IgM^+^IgD^+^ B cells were analyzed by nested PCR using forward Iμ and reverse Cδ or Sγ1 primers followed by Southern-blotting using indicated specific probes. Data are from three independent experiments. **b** Expression of *RAD52* and *AICDA* transcripts was analyzed 48 h after *RAD52* siRNA or Scra siRNA transfection by qRT-PCR and normalized to *HPRT* expression. Data are mean ± SEM of three independent experiments. **p < 0.01, ns: not significant (unpaired two-tailed *t*-test). **c** Expression of RAD52 and AID proteins were analyzed 72 h after *RAD52* siRNA or Scra siRNA transfection by specific Western blotting. Data are representative of three independent experiments. **d** Expression of *V*_*H*_*DJ*_*H*_*−Cδm* and *V*_*H*_*DJ*_*H*_*−Cδs* transcripts as analyzed by semi-quantitative RT-PCR with of serial two-fold dilution of cDNA templates. Data are representative of three independent experiments. **e** RAD52 is recruited to σδ region DNA in human B cells undergoing CSR to IgD. Recruitment of RAD52 to Sμ and σδ region DNA in human blood naive IgM^+^IgD^+^ B cells stimulated for 120 h with CpG plus IL-2 and IL-21, as analyzed by specific ChIP-qPCR. Data are mean ± SEM of three or four independent experiments. *p < 0.05, **p < 0.01 (unpaired two-tailed *t*-test). Source data are provided as a Source Data file.

Next, we addressed the expression of mIgD and sIgD and its regulation by stimuli inducing CSR to IgD. Resting naïve IgM^+^IgD^+^ B cells expressed mIgD and mIgM, but virtually no sIgD or sIgM, reflecting high levels of *V*_*H*_*DJ*_*H*_*−Cδm* and *V*_*H*_*DJ*_*H*_*−Cμm* trancripts and low levels of *V*_*H*_*DJ*_*H*_*−Cδs* and *V*_*H*_*DJ*_*H*_*−Cμs* transcripts (Fig. 7a). Induction of CSR to IgD (by LPS or CD154 plus IL-4) resulted in loss of mIgD, emergence of *V*_*H*_*DJ*_*H*_*−Cδs* transcripts together with *V*_*H*_*DJ*_*H*_*−Cμs* transcripts and significant IgD secretion (Fig. 8a, b). By contrast, application of IgD CSR non-inducing stimuli (LPS plus TGF-β and RA) to similar naive IgM^+^IgD^+^ B cells resulted in increased mIgD, no change in *V*_*H*_*DJ*_*H*_*−Cδm* transcripts, and marginal IgD secretion (Fig. 7a, b, Supplementary Fig. 7b). The changes in *V*_*H*_*DJ*_*H*_*−Cδm, V*_*H*_*DJ*_*H*_*−Cδs* transcripts, mIgD and sIgD brough about by IgD CSR-inducing stimuli paralleled the downregulation of *Zfp318* transcripts and Zfp318 protein—Zfp318 represses the TTS that mediates alternative transcriptional *V*_*H*_*DJ*_*H*_*−Cμ/V*_*H*_*DJ*_*H*_*−Cδ* termination, thereby allowing for long-range transcription throughout *V*_*H*_*DJ*_*H*_*−Cμ-s-m-Cδ-s-m* DNA (Fig. 7c–e and Supplementary Figs. 7c, 8a). *Zfp318* downregulation by LPS plus IL-4 was associated with reduced histone H3K27me1 (Supplementary Fig. 8b), an epigenetic activation mark, at the *Zfp318* gene locus. Zfp318 downregulation was specific to IgD CSR, as it did not occur in response to IgA CSR-inducing stimuli (LPS plus TGF-β and RA). *ZFP318* downregulation concomitant with decreased mIgD expression and increased IgD secretion was reproduced in human B cells submitted to IgD CSR-inducing stimuli (CpG plus IL-2 and IL-21) but not IgD CSR non-inducing stimuli (CpG plus IL-4 and IL-21) (Fig. 7f). Similarly, *ZFP318* transcripts and ZFP318 protein were downregulated in human B cells undergoing IgD CSR in vivo, as in peripheral blood and tonsils (Fig. 7g, h). Zfp318 downregulation was independent of and likely preceded expression of AID or Rad52, as revealed by virtual absence of *Zfp318* transcripts in LPS plus IL-4-induced *Aicda*^*−/−*^ B cells, *Rad52*^*−/−*^ B cells, and *Rad52*^*+/+*^ B cells, all of which lost mIgD expression as compared to similar B cells stimulated by IgA CSR-inducing stimuli (LPS plus TGF-β and RA) (Fig. 7i–k, Supplementary Fig. 7d). Thus, the stimuli that specifically induce CSR to IgD downregulate ZFP318/Zfp318 independently of AID or Rad52 expression and prior to Sμ–σδ DNA recombination.

### RAD52 knockdown reduces Sμ–σδ DNA recombination and IgD secretion in human B cells

The high frequency of microhomologies in Sμ–σδ junctions of human tonsil B cells in vivo and human B cells induced to undergo CSR to IgD in vitro (Fig. 3a and Supplementary Figs. 1, 4) suggested to us that RAD52 also mediates Sμ–σδ DNA recombination in human B cells. We purified naïve IgM^+^IgD^+^ B cells from peripheral blood of three healthy subjects and knocked down using *RAD52*-specific siRNAs *RAD52* transcripts and RAD52 protein by up to 75% and 95%, respectively. In these B cells, Sμ–σδ recombination, as induced by CpG plus IL-2 and IL-21, was virtually abolished, while *AICDA* or AID expression and Sμ–Sγ1 recombination were not altered (Fig. 8a–c). The reduced Sμ–σδ DNA recombination in RAD52 knockdown human B cells was associated with decreased expression of *V*_*H*_*DJ*_*H*_*−Cδs* transcripts, without significant alteration of *V*_*H*_*DJ*_*H*_*−Cδm* transcripts (Fig. 8d). The critical role of RAD52 in human CSR to IgD was emphasized by RAD52 recruitment to Sμ and σδ regions in human naïve IgM^+^IgD^+^ B cells induced to undergo CSR to IgD (by CpG plus IL-2 and IL-21) in vitro, human tonsil (IgD^+^) B cells undergoing CSR to IgD in vivo, but not in unstimulated naïve IgD^+^IgM^+^ B cells (Fig. 8e). Thus, Rad52 critically mediates CSR to IgD through Sμ–σδ recombination in human B cells.

### Sμ–σδ DNA recombination leads to IgD plasma cell differentiation

**Fig. 9:**
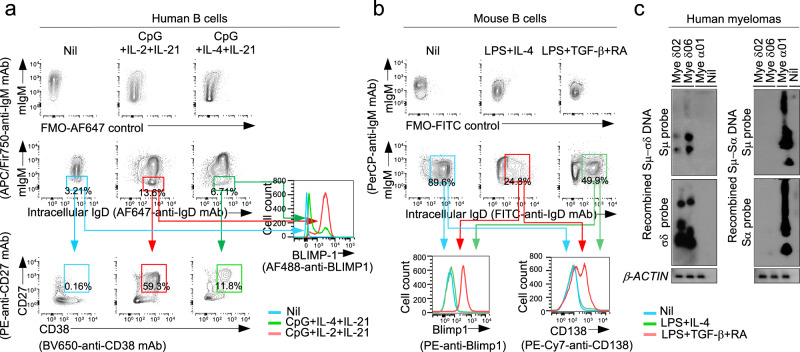
B cells undergoing CSR to IgD differentiate to IgD-producing plasmablasts/plasma cells. **a** Human blood naive IgM^+^IgD^+^ B cells were stimulated with CpG plus IL-2 and IL-21, which induce IgD CSR, or CpG plus IL-4 and IL-21, which do not induce IgD CSR. Proportions of CD138^+^IgM^−^IgD^*+*^ plasmablasts/plasma cells among intracellular sIgM^−^IgD^*+*^ B cells and BLIMP-1 expression in intracellular sIgM^*–*^IgD^*+*^ cells, as analyzed 120 h post-stimulation by flow cytometry. Alexa Fluor 647-fluorescence minus one (FMO) controls are shown as reference. **b** Mouse *Rad52*^*+/+*^ B cells and *Rad52*^*−/−*^ B cells stimulated with LPS plus IL-4, which induce IgD CSR. Proportions of sCD138^+^ plasmablasts/plasma cells among intracellular IgD^*+*^sIgM^*−*^ cells and Blimp-1 expression in intracellular IgD^*+*^sIgM^−^ cells, as analyzed 96 h post-stimulation by flow cytometry. FITC-FMO controls are shown as reference. Data in **a** and **b** are representative of three independent experiments. **c** Recombined Sμ–σδ and Sμ–Sα DNA in two IgD^+^ myelomas and one IgA^+^ myeloma, as analyzed by specific nested PCR followed by Southern-blotting using indicated probes.

To determine whether the substantial IgD secretion we observed upon induction of CSR to IgD (Figs. 4b, 5c–h, 7b, f) reflected increased plasma cell differentiation, we analyzed human naïve IgM^*+*^IgD^+^ B cells induced to undergo CSR to IgD by CpG plus IL-2 and IL-21. More than 13% of these B cells became mIgM^−^ and intracellular IgD^+^ compared to 6.8% of their counterparts stimulated by CpG plus IL-4 and IL-21, not undergoing to CSR to IgD (Fig. 9a, Supplementary Fig. 9a). More than 90% of the IgM^−^IgD^+^ B cells emerging from CpG plus IL-2 and IL-21 simulation expressed BLIMP-1 and almost 60% were CD27^+^CD38^+^ versus about 10% of IgM^−^IgD^+^ B cells from CpG plus IL-4 and IL-21 stimulation expressing BLIMP-1 and <12% being CD27^+^CD38^+^. Among mouse naïve IgM^*+*^IgD^+^ B cells induced to undergo CSR to IgD by LPS plus IL-4, about 25% expressed intracellular IgD. All these B cells also expressed Blimp-1 and 70% or more acquired CD138 (Fig. 9b and Supplementary Fig. 9b). By contrast, among naïve IgM^*+*^IgD^+^ B cells induced to undergo CSR to IgA (by LPS plus TGF-β and RA), about 50% expressed intracellular IgD and mIgD. Virtually none of these IgD^+^ B cells, however, expressed Blimp-1 or acquired surface CD138 (Figs. 7a, 9b). The relevance of IgD CSR to plasma cell differentiation and sustained IgD secretion was furthered by analysis of three human myelomas, two IgD and one IgA. Both IgD myelomas displayed Sμ–σδ DNA, but not Sμ–Sα DNA recombination (Fig. 9c). Conversely, the IgA myeloma showed Sμ–Sα, but not Sμ–σδ DNA recombination. Thus, IgD^+^ B cells emerging by CSR would be prone to differentiate into IgD-secreting plasmablasts/plasma cells for sustained IgD secretion. And such IgD^+^ B cells may function as precursors of neoplastic IgD^+^ transformants.

### B cell Rad52 phosphorylation, increased CSR to IgD and IgD autoantibodies in systemic autoimmunity

**Fig. 10:**
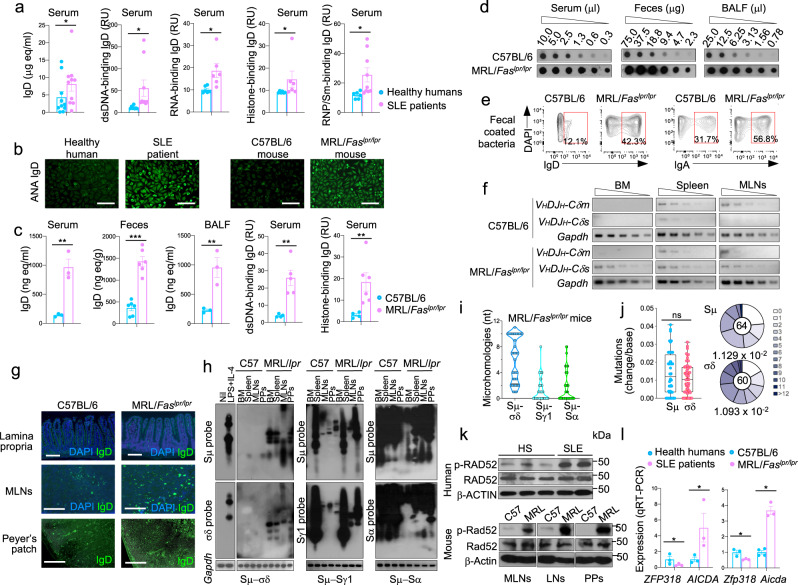
p-Rad52 expression, CSR to IgD and antinuclear antigen IgD autoantibodies in lupus patients and mice. **a** Serum total and double-strand DNA (dsDNA)-, RNA-, histone-, or RNP/Sm-binding IgD in healthy human subjects and systemic lupus erythematosus (SLE) patients, as analyzed by specific ELISAs. Each dot represents the datum from one individual human subject. Mean ± SEM of 6–10 healthy subjects or SLE patients are depicted. *p < 0.05 (unpaired two-tailed *t*-test). **b** Human and mouse antinuclear autoantibodies (ANAs), as visualized by indirect immunofluorescence microscopy on HEp-2 cells that were incubated with serum from a healthy human subject, an SLE patient, a C57BL/6 mouse, or a MRL/*Fas*^*lp/lpr*^ mouse, as revealed by FITC-labeled rat mAb to human or mouse IgD. Scale bar = 50 μm. **c** Total IgD in serum, feces and BALF as analyzed by dot-blotting, and concentrations of IgD autoantibodies to dsDNA or histone in serum of C57BL/6 and MRL/*Fas*^*lpr/lpr*^ mice, as analyzed by specific ELISAs. Each dot represents datum from one individual mouse. Data are mean ± SEM of 3–9 mice, as indicated. **p < 0.01, ***p < 0.001 (unpaired two-tailed *t*-test). **d** IgD concentrations in serum, feces and BALF from C57BL/6 and MRL/*Fas*^*lpr/lpr*^ mice, as analyzed by dot-blots. Shown are dot-blots from one C57BL/6 and one MRL/*Fas*^*lpr/lpr*^ mouse, representative of 3–9 C57BL/6 and MRL/*Fas*^*lpr/lpr*^ mice. **e** Bacteria-bound IgD and IgA in feces from C57BL/6 and MRL/*Fas*^*lpr/lpr*^ mice, as analyzed by flow cytometry. **f** Expression of *V*_*H*_*DJ*_*H*_*−Cδm, V*_*H*_*DJ*_*H*_*−Cδs, V*_*H*_*DJ*_*H*_*−Cμm*, and *V*_*H*_*DJ*_*H*_*−Cμs* transcripts in bone marrow (BM), spleen and mesenteric lymph nodes (MLNs), as analyzed semi-quantitative RT-PCR by serial two-fold dilutions of cDNA templates. Shown are RT-PCR data from one C57BL/6 mouse and one MRL/*Fas*^*lpr/lpr*^ mouse, representative of three C57BL/6 and three MRL/*Fas*^*lpr/lpr*^ mice. **g** IgD^+^ B cells in lamina propria, MLNs and Peyer’s patches (PPs) of C57BL/6 and MRL/*Fas*^*lpr/lpr*^ mice, as visualized by fluorescent microscopy. Scale bar =  100 μm. **h** Recombined junctional Sμ–σδ, Sμ–Sγ1, and Sμ–Sα DNAs in bone marrow, spleen, MLNs, and PPs B cells from C57BL/6 and MRL/*Fas*^*lpr/lpr*^ mice as analyzed by specific nested PCR using forward Iμ and reverse Cδ, Sγ1 or Sα primers, followed by Southern-blotting using indicated probes. Data are representative of three independent experiments. **i** Sμ–σδ, Sμ–Sγ1 and Sμ–Sα junctional DNAs in non-immunized MRL/*Fas*^*lpr/lpr*^ mice, as amplified by nested PCR and sequenced by MiSeq. The length and numbers of nucleotide overlaps (microhomologies) in Sμ–σδ, Sμ–Sγ1, and Sμ–Sα junctional DNAs are rendered by violin plots. Each dot represents a unique sequence (*n* = 45 per group). **j** Somatic point-mutations in Sμ and σδ regions abetting recombined Sμ−σδ DNA junctions in IgD class-switched spleen B cells from three MRL/*Fas*^*lpr/lpr*^ mice. Mutations were identified in a 48–506 nt stretch of Sμ or σδ regions in unique Sμ–σδ DNA recombination sequences. Each dot represents an individual sequence. ns: not significant (unpaired two-tailed *t*-test). Box and whiskers plots show the median, quartiles, maximum, and minimum of mutation frequencies in Sμ and σδ regions. In pie charts, the size of slices denotes the proportion of sequences with the same number of mutations and the gray hue denotes the number of point-mutations per sequence. Center of pie shows the total number of independent sequences analyzed. Below the pie charts is the overall mutation frequency (change/base). **k** Expression of phosphorylated Rad52 (p-Rad52), Rad52 and β-Actin proteins in peripheral blood B cells from healthy human subjects and SLE patients as well as B cells from C57BL/6 mice and MRL/*Fas*^*lpr/lpr*^ mice, as analyzed by specific Western blotting using rabbit anti-p-Rad52 Ab or anti-β-Actin mAb—p-Rd52 (Y104) Ab detected endogenous levels of Rad52 protein only when phosphorylated at tyrosine 104. **l** Expression of *ZFP318* and *AICDA* transcripts in B cells from healthy human subjects and SLE patients (left panel), as well as *Zfp318* and *Aicda* transcripts in MLNs from C57BL/6 and MRL/*Fas*^*lpr/lpr*^ mice (right panel), as analyzed by specific qRT-PCR. Data are mean ± SEM of three healthy human subjects, three SLE patients, three C57BL/6 mice, and three MRL/*Fas*^*lpr/lpr*^ mice. *p < 0.05 (unpaired two-tailed *t*-test). No adjustments were made for multiple comparisons. Source data are provided as a Source Data file.

Serum IgD have been suggested to increase in patients with inflammatory autoimmune diseases, such as SLE^29^ and rheumatoid arthritis^30^, and in hereditary autoinflammatory syndromes, most notably the hyper-IgD syndrome (HIDS)^31–34^. In healthy humans, many circulating B cells make IgD that react with self-antigens including nuclear components, such as DNA^35^. We found SLE patients to display significantly higher levels of circulating IgD, including IgD specific for nuclear antigens, than their healthy subject controls (Fig. 10a, b). Similarly, we found lupus-prone MRL/*Fas*^*lpr/lpr*^ mice to display higher levels of total IgD and/or IgD antinuclear autoantibodies than their wildtype C57BL/6 mouse counterparts in serum, feces and BALF as well as increased IgD-coated bacteria in feces (Fig. 10b–e). Overall, this was a reflection of high levels of *V*_*H*_*DJ*_*H*_*−Cδs* transcripts in bone marrow, spleen, MLNs and Peyer’s patches B cells as well as increased numbers of IgD^+^ B cells in lamina propria, MLNs and Peyer’s patches (Fig. 10f, g and Supplementary Fig. 9c). In MRL/*Fas*^*lpr/lpr*^ mice, the elevated IgD levels reflected the increased numbers of IgD-producing cells and increased B cell Sμ–σδ DNA recombination in bone marrow, spleen, MLNs and Peyer’s patches (Fig. 10h). Increased CSR to IgD in MRL/*Fas*^*lpr/lpr*^ mice was characterized by greater frequency and length of microhomologies in Sμ–σδ as compared to Sμ–Sγ1 and Sμ–Sα junctional sequences, as well as a high frequency of somatic point-mutations in areas abetting Sμ-Sδ DNA junctions (Fig. 10i, j and Supplementary Fig. 10). In both SLE patients and lupus MRL/*Fas*^*lpr/lpr*^ mice, the high level of CSR to IgD, total IgD and IgD nuclear autoantibodies likely stemmed from the higher level of B cell Rad52 and/or p-Rad52 (Fig. 10k). Further, consistent with the high levels of Rad52 and/or p-Rad52 expression and CSR to IgD, expression of *ZFP318/Zfp318* was decreased in B cells of such SLE patients and lupus MRL/*Fas*^*lpr/lpr*^ mice (Fig. 10l). Thus, B cells expressing high levels of phosphorylated Rad52 would underpin the high levels of IgD and IgD autoantibodies to nuclear antigens in SLE patients and in lupus MRL/*Fas*^*lpr/lpr*^ mice. As shown in these mice, Sμ–σδ DNA recombination events involving high frequency of junctional microhomologies occur in circulating B cells and B cells in different body districts, including the respiratory tract, gut lymphoid formations, spleen and splanchnic draining lymph nodes, giving rise to high levels of IgD autoantibodies locally and systemically.

## Discussion

The mechanism of CSR to IgG, IgA, and IgE is quite well understood, as mediated by Ku70/Ku86-dependent NHEJ, although occurrence of a “residual” IgM to IgG CSR in B cells lacking Ku70/Ku86 expression has suggested the existence of a Ku70/Ku86-independent A-EJ synaptic mechanism^18–20^. Mice lacking 53BP1, in which NHEJ-dependent CSR to IgG, IgA and IgE was significantly decreased—53BP1 protects resection of DSB ends, thereby skewing the synaptic process toward NHEJ—showed increased CSR to IgD and increased circulating IgD levels^22,23^, suggesting that the short-range Sμ–σδ CSR is mediated by a 53BP1-independent synaptic process involving resected DSB ends and entailing a high frequency of Sμ–σδ junctional microhomologies. This together with our previous demonstration that Rad52 plays a central role in synapsing intra-Sμ region resected DSB ends as well as *c-Myc/IgH* locus translocations also involving resected DSB ends—both processes entailing significant junctional microhomologies—prompted us to hypothesize that Rad52 synapses Sμ and σδ DSB involving complementary overhangs for CSR to IgD^20^. Indeed, here we showed that Rad52 effects CSR to IgD through a MM A-EJ mechanism in concert with Zfp318 modulation of primary *V*_*H*_*DJ*_*H*_*−Cμ-s-m-Cδ-s-m* transcription in mouse and human B cells (Supplementary Fig. 11), thereby unveiling a previously unknown and critical role of the HR factor Rad52 in a specific mammalian DNA repair process.

We have provided here unequivocal evidence that Rad52 is critical for CSR to IgD in vitro and in vivo, in mouse and human B cells. Sμ–σδ DNA recombination was ablated in mouse *Rad52*^*−/−*^ B cells and virtually abrogated in human *RAD52* knockdown B cells—as expected^1,5^, Sμ–σδ DNA recombination could not occur in the absence of AID. AID introduces DSBs in σδ as it does in Sμ, Sγ, Sα or Sε. In both mouse *Rad52*^*−/−*^ B cells and human *RAD52* knockdown B cells, ablation or virtual ablation of post-recombination *V*_*H*_*DJ*_*H*_*−Cδs* transcripts resulted in reduced IgD secretion, which occurred in presence of unaltered levels of transmembrane *V*_*H*_*DJ*_*H*_*−Cδm* transcripts, at least within the first 72 h from CSR induction. Interestingly, the stimuli that selectively induced CSR to IgD modulated the overall levels of *Ku70/KU70*, *Ku86/KU86*, and *Rad52/RAD52* transcripts while significantly upregulating *Aicda/AICDA* in both mouse and human B cells. This was concomitant with induction of AID and moderate decrease in Rad52 protein, which, in fact, was increasingly phosphorylated at Tyr104. Rad52 Tyr104 phosphorylation boosts Rad52-mediated DNA single-strand annealing and is possibly effected by c-ABL kinase^28^. In human and mouse B cells, Rad52 phosphorylation was promoted by IgD CSR-inducing stimuli. Rad52 involvement in CSR to IgD was further emphasized by recruitment of this protein to the σδ region (in addition and necessarily to Sμ) in vivo in human tonsil IgD^+^ B cells, as well as in vitro, in mouse and human naïve B cells induced to undergo CSR to IgD, but no or only marginally in similar B cells undergoing CSR to IgG3 or IgA. Instead, these B cells recruited Ku70/Ku86 to Sγ3 and Sα, consistent with a dominant contribution of NHEJ to CSR to IgG3 and IgA.

Rad52 is a member of the eponymous epistasis group for DSB repair that shows strong evolutionary conservation^17,36^. In *Saccharomyces cerevisiae*, Rad52 is a key element of the HR pathway, and its deletion or mutation impairs DNA DSB repair^37,38^. Indeed, yeast Rad52 is a DSB recombination mediator and a facilitator of annealing of complementary DNA single-strands^39,40^. As a cofactor of Rad51, it can form nucleoprotein filaments with single-strand DNA and promotes strand pairing by overcoming the inhibitory effect of replication protein A (RPA)^20,41^. Rad52 mutation or even deletion results in no obvious abnormalities in viability or functions in mammalian cells. As we have shown, *Rad52*^*−/−*^ mice displayed no obvious alteration of immune system elements, including B cells^20^, possibly owing to the presence of mammalian gene paralogues, such as *BRCA2* and *RAD51*, which by encoding functions related to Rad52, can compensate for the absence of this HR factor^42^. Human BRCA2 functions as a recombination mediator by facilitating RAD51 nucleoprotein filament formation^40,43–45^. Nevertheless, human BRCA2 cannot facilitate annealing of RPA-coated DNA, a function that Rad52 carries out efficiently in the absence of BRCA2^46^. This together with Rad52 involvement in DSB repair at stalled or collapsed replication forks points at a unique role of this HR factor in catalyzing single-strand annealing in homology-directed DNA repair in human cells^47–49^. Indeed, in these cells, Rad52 would mediate synapses of intra-V gene segment resected DSBs during SHM^50^.

Our identification of Rad52 as essential in the IgD CSR Sμ–σδ synaptic process provides, to the best of our knowledge, the first demonstration of a critical and dedicated role of this factor in mammalian DNA DSB repair. The short-range Rad52-mediated Sμ–σδ recombination of resected DSB ends adds to the other Rad52-mediated short-range DSB recombination we recently uncovered: intra-Sμ region DSB recombination^20^. This, like Sμ−σδ synapsing, entails insertion of significant junctional nucleotide microhomologies^20^. In this function, as in CSR to IgD, Rad52 is not fungible in mouse or human B cells. The identification of Rad52 as the critical element in Sμ–σδ synapsis sheds light on the mechanistic nature of the CSR microhomology-mediated A-EJ DSB repair pathway, originally referred to as A-NHEJ^19^. As per our current findings, the CSR A-EJ pathway uses HR Rad52 to synapse upstream and downstream DSB overhangs through MMEJ, hence our definition of this process as MM A-EJ. This would not require a homologous template as a guide, as the HR pathway does. Although DNA polymerase θ has been suggested to contribute to A-EJ^21^, our previous findings do not support a role of this polymerase in Rad52-mediated intra-Sμ DSB recombination or Sμ−Sγ1, Sμ−Sγ3, Sμ−Sγ2a/Sγ2c, and Sμ−Sα recombination (CSR to IgG1, IgG3, IgG2a/IgG2c, and IgA)^20^. While Rad52 MM A-EJ functions as a backup pathway for CSR in B cells defective in NHEJ, it also synapses DSB ends in presence of a full complement of NHEJ elements^51^, as exemplified by microhomologies in S–S junctions in a minor proportion of B cells that class-switched to IgG, IgA and IgE, and the disappearance of such microhomologies upon Rad52 ablation^20^.

As we showed here, Rad52 works in concert with Zfp318 to modulate IgD expression through an interplay of alternative RNA splicing and DNA recombination, the latter after AID intervention. Zfp318 represses the TTS intercalated between Cμ and Cδ exons within the *Ighμ/Igh*δ loci transcriptional complex unit^10,11^, thereby allowing for expression of long primary *V*_*H*_*DJ*_*H*_*-Cμ-s-m-Cδ-s-m* RNA. Zfp318, however, also simultaneously allows for the continuous expression of primary *Iμ-Cμ-s-m-Cδ-s-m* RNA transcripts. In fact, albeit possibly more abundant, hence their predominant detection in our specific PCR assays, *Iμ-Cδ* transcripts (in secretory or membrane form) are identical in sequence to their post-recombination *Iμ-Cδ* counterparts (Fig. 1a, b). During B cell development, Zfp318 expression closely parallels mIgD expression^10,11^. And, consistent with its repression of the TTS intercalated between the *Cμ* and *Cδ* exons complex, the Zfp318 protein is expressed during the transition from immature IgM^+^IgD^−^ to mature IgM^+^IgD^hi^ B cell^10,11^. As we showed here, naïve mature B cells which express high levels of mIgD also express high levels of *Zfp318* transcripts and Zfp318 protein. In these B cells, stimuli that induced Sμ–σδ DNA recombination, yielding primary *V*_*H*_*DJ*_*H*_*-Cδ-s-m* RNA transcripts, induced profound downregulation of *Zfp318* transcripts and Zfp318 protein, suggesting that relieving Zfp318-mediated TTS repression is a prerequisite for Sμ–σδ DNA recombination to unfold. Conversely, as we also showed here, naïve mature IgM^+^IgD^+^ B cells submitted to stimuli that induced CSR to isotypes other than IgD, such as IgA (by LPS plus IL-4 and RA), further upregulated *Zfp318* transcripts and Zfp318 protein, concomitant with no Sμ–σδ recombination, but rather allowing for massive expression of mIgD rather than sIgD.

The role of Zfp318 as gene transcription regulator is highly specific for IgD, as genome-wide transcriptome analysis of B cell Zfp318-deficient (*Vav-Cre* dependent deletion) mice identified *Sva* as the only other gene altered in expression^11^ – interestingly, *Sva* is also involved in alternative splicing, albeit outside the *IgH* locus^52^. Zfp318 would be under the control of 5'AMP-activated protein kinase (Ampk). This is phosphorylated by Lbk1^53^, whose signaling triggers the B cell GC reaction. Indeed, Lbk1’s failure to activate Ampk or Ampk loss specifically muted Zfp318 expression and IgD transcription^53^. In contrast, activation of Ampk by phenformin impaired GC formation^53^, likely by heightening Zfp318 expression and possibly in addition to other mechanisms. This would result in increased expression of primary *V*_*H*_*DJ*_*H*_*−Cμ-s-m-Cδ-s-m* RNA transcripts but not Sμ–σδ DNA recombination, suggesting that CSR to IgD is one of the multiple and complex events inherent to GC formation. This is generally triggered by naturally occurring, generally, microbial stimuli, as in tonsil GCs and GCs or other secondary lymphoid formations in aerodigestive mucosae^1,5,6^. Consistent with the contention that Ampk mediates the regulation of Zfp318 as well as the contrasting impact of IgD CSR-inducing (LPS plus IL-4) and non-inducing stimuli (LPS plus TGF-β and RA) on expression of Zfp318, stimulation of both human and mouse cells by LPS has been shown to result in dephosphorylation/inactivation of Ampk, while similar cell stimulation by TGF-β would result in rapid phosphorylation/activation of this protein kinase^54^.

In our experiments, stimuli that induced CSR to IgD (e.g., LPS plus IL-4 in mouse B cells, and CD154 plus IL-2 and IL-21 in human B cells) also downregulated Zfp318 expression which, in turn, reduced *V*_*H*_*DJ*_*H*_*−Cδm* transcript level and mIgD, while greatly increasing *V*_*H*_*DJ*_*H*_*−Cδs* transcripts and sIgD. This argues for CSR to IgD to be critical for significant IgD secretion. Indeed, stimuli that induced Sμ–σδ recombination and IgD secretion also induced plasmablast/plasma cell differentiation, as shown by Blimp-1, CD38, and CD27 expression in human B cells, and Blimp-1 and CD138 expression in mouse B cells. A similar outcome was not produced by stimuli that did not induce Sμ–σδ recombination and IgD secretion in both mouse and human B cells. Thus, while alternative splicing of long primary *V*_*H*_*DJ*_*H*_*−Cμ-s-m-Cδ-s-m* RNA transcripts in B cells that have not undergo CSR would make some contribution to the overall level of IgD production in vivo, CSR to IgD would be required for substantial and sustained IgD production, as secreted by plasmablasts/plasma cells or by neoplastic transformants, such as IgD myeloma cells. The limited IgD amounts detected in supernatants of mouse or human B cells primed by stimuli that induced high levels of mIgD but not Sμ–σδ synaptic recombination would result from translation of alternative spliced long primary *V*_*H*_*DJ*_*H*_*−Cμ-s-m-Cδ-s-m* RNA transcripts as well as some “shedding” of mIgD.

Bacteria and viruses have been suggested to play an important role in driving CSR to IgD, generally through stimulation of TLRs in gut and respiratory lymphoid tissues, and mesenteric lymph nodes, possibly leading to emergence of plasmablasts and plasma cells secreting IgD^1,3–5,52,55–57^. Circulating IgD are increased in HIV patients with frequent respiratory infections, and in mice undergoing acute viral infections, suggesting a role for IgD in airways mucosae antimicrobial protection^1,3,5,55,57,58^. In the in vivo T-dependent antibody response to OVA, ablation of Rad52 (*Rad52*^*−/−*^ mice) resulted in reduced levels of total and specific IgD not only in circulating blood but also in total and/or bacteria-bound IgD in feces as well as decreased numbers of IgD^+^ B cells in lamina propria and MLNs, a privileged site of IgD CSR^12^. In these mice, the marginal level of IgD in BALF suggests that in the lack of CSR, the compensatory expression of IgD by alternative splicing contributes to overall IgD levels systemically and in the gut but only minimally in respiratory mucosae, where the level of IgD is would result almost exclusively from CSR. As predicted by our previous findings^20^, the decreased IgD levels in *Rad52*^*−/−*^ mice were associated with increased IgG1 and IgA as well as greatly reduced microhomologies in Sμ–Sγ1 and Sμ–Sα junctions. This reflected the lack of Rad52 contribution to the synaptic process underpinning such junctions as well as the lack of Rad52 competition with Ku70/Ku86 for binding to DSB free ends^20^, resulting solely in Ku70/Ku86-mediated NHEJ, a process that limits microhomologies to 0–3 nt^16^.

Information on the contribution of IgD to autoimmune responses is scant and contradictory. Self-antigen-binding and mostly polyreactive IgD occur in healthy subjects, much like IgM or even IgG and IgA do^35,59–62^. Interestingly, in healthy humans, circulating mature B cells class-switched to IgD have been found to make IgD reactive with self-antigens, including nuclear antigens, such as single-strand DNA and double-strand DNA^35^. High levels of IgD have been reported in rheumatoid arthritis patients and thought to possibly be implicated in the pathogenesis of the disease^30^. IgD expression, however, has been speculated to exert an inhibitory effect on B cell autoreactivity, as suggested by elevated autoantibody production, increased deposition of immune complexes in kidneys, and severe nephritis in lupus-prone C56BL/6*lpr* mice with deletion of the *Igδ* locus^63,64^. Our findings showed total and self-reactive IgD (dsDNA, histone, RNP/Sm or RNA and ANAs) to be elevated in the circulation of SLE patients and lupus-prone MRL/*Fas*^*lpr/lpr*^ mice. The latter displayed higher levels of IgD in serum, BALF, and feces, than their wildtype C57BL/6 counterparts. Such high IgD levels reflected CSR recombinations that included Sμ–σδ junctions with extensive microhomologies and high frequency of somatic mutations in the DNA areas abetting Sμ–σδ junctions. IgD CSR occurred in different districts, such as bone marrow, spleen, MLNs, and Peyer’s patches, and was reflected in IgD^+^ B cells in those districts. This together with high B cell levels of phosphorylated Rad52 and low levels Zfp318 indicated that in murine and human lupus, IgD autoantibodies stem from extensive B cell Sμ–σδ recombination, as also supported by high levels of AID, rather than Zfp318-dependent alternative splicing of primary *V*_*H*_*DJ*_*H*_*−μ-s-m-Cδ-s-m* RNA transcripts. Our findings do not suggest a “protective” role of IgD in autoimmunity^63,64^, while supporting a role of CSR to IgD in systemic lupus autoantibody responses.

Collectively, our data outline a critical and dedicated role of Rad52 in mediating the synapsis of Sμ with σδ DSB resected ends. They also provide the first demonstration of Rad52 as essential element of the poorly understood A-EJ process underpinning resolution of resected end DSBs in B cell *IgH* locus. In malignant B cells, Rad52 is involved in DNA recombination events that give rise to DNA deletions and translocations. As we previously showed, Rad52 ablation reduced the frequency of *c-Myc/IgH* translocations in mouse *p53*^−/−^ B cells by >70%, with the residual translocations containing limited microhomologies^20^. Whether Rad52 intervention extends to other modalities of A-EJ in neoplastic and non-neoplastic lymphoid mammalian cells remains to be determined. The importance of the Rad52 function newly unveiled here is further emphasized by our demonstration that this highly conserved HR element is critical for CSR to IgD in not only mouse but also human B cells. This together with the further reduction of the physiologically moderate microhomologies in Sμ–Sγ1, Sμ–Sγ3, Sμ–Sα, and Sμ–Sε junctions in *Rad52*^*−/−*^ B cells (current data and refs. ^18–20^) solidifies the role of Rad52 as critical mediator of the A-EJ backup pathway underpinning the residual CSR to IgG, IgA and IgE in the absence of Ku70/Ku86 proteins^20^. Our findings also showed how stimuli that induce Sμ–σδ recombination coordinate Rad52 function, as enabled by phosphorylation, with downregulation of Zfp318, unique repressor of the TTS intercalated between the Cμ and Cδ loci, whose activity allows transcription throughout *V*_*H*_*DJ*_*H*_*−Sμ-Cμ-s-m-σδ-Cδ-s-m* and *Iμ-Cμ-s-m-*σ*δ-Cδ-s-m*. Further, they indicate that CSR to IgD is required for sustained IgD secretion and, possibly, a prerequisite for IgD plasma cell differentiation. They also add new and significant information to a potential role of CSR to IgD, as promoted by Rad52 phosphorylation, in systemic autoimmunity. Finally, they would provide important new molecular information to approach the virtually unexplored mechanistic underpinning of hyper-IgD syndrome, a relatively rare but a severe autoinflammatory disease associated with mevalonate kinase deficiency (due to *MVK* recessive mutations) and exorbitant levels of IgD^31,32,65^.

## Methods

### Mice

*Rad52*^*−/−*^ mice were generated by replacing exon 3 of the *Rad52* gene with positive selection marker neomycin, as driven by the phosphoglycerate kinase (PGK) promoter, and an upstream mouse sequence functioning as a transcription terminator (Dr. Albert Pastink, Leiden University, Leiden, The Netherlands)^36^. *Rad52*^*−/−*^ mice were backcrossed to C57/BL6 mice for more than six generations. No full length or truncated Rad52 protein was produced from the disrupted allele^36^. *Rad52*^*−/−*^ mice were viable and fertile, and showed no gross abnormalities. *Aicda*^*−/−*^ mice (C57BL/6 background)^66^ were obtained from Dr. Tasuku Honjo (Kyoto University, Kyoto, Japan). C57BL/6 mice (Stock # 000664) and MRL/*Fas*^*lpr/lpr*^ mice (Stock # 000485) were purchased from Jackson Laboratory (Bar Harbor, Maine). For OVA immunization, *Rad52*^*+/+*^ and *Rad52*^*−/−*^ mice were injected intraperitoneally with 20 μg of OVA in 100 μl alum (Imject Alum, Pierce) i.p., three times, at day 0, 7 and 14. One week after the last injection, the mice were euthanized using a euthanasia CO_2_ chamber (filled at a rate of 20–30% CO_2_ chamber volume per minute, which has been shown to cause the least amount of distress to rodents) for ex vivo analysis. All mice were housed in the Specific Pathogen-Free/SPF facility of the University of Texas Health Science Center at San Antonio. Both male and female mice aged 8–12 weeks were used for the experiments. The Institutional Animal Care and Use Committees (IACUC) of the University of Texas Health Science Center at San Antonio approved all animal protocols.

### Mouse B cells and CSR induction in vitro

Naïve IgM^+^IgD^+^ B cells were isolated from spleens of 8–12-week-old C57BL/6, *Rad52*^*−/−*^ or *Aicda*^*−/−*^ mice, as described^25^. B cells were resuspended in RPMI 1640 medium with 10% FBS (FBS-RPMI), 50 mM β-mercaptoethanol and 1x antibiotic-antimycotic mixture (15240-062; Invitrogen) and stimulated with LPS (4 μg/ml) from *Escherichia coli* (055:B5; Sigma-Aldrich), CD154 (1 U/ml; obtained from membrane fragments of baculovirus-infected Sf21 insect cells^25^), CpG ODN 1826 (1.0 μM; Custom synthesized by Eurofins Genomics) or R848 (1.0 μM; Medkoo) plus nil, IL-4 (5.0 ng/ml; R&D Systems) and/or TGF-β (2.0 ng/ml; R&D Systems) and retinoic acid (RA, 10 nM) or anti-BCR Ab (anti-δ mAb-dextran, 30 ng/ml; Fina Biosolutions). Mouse B cells were cultured in FBS-RPMI at 37 °C in 48-well plates for 24, 48, 72 and 96 h.

### Human B cells and CSR induction in vitro

Naïve IgM^+^IgD^+^ B cells were purified by negative selection using the EasySep^TM^ human naive IgM^+^IgD^+^ B cell enrichment kit (19254; StemCell Technologies), following manufacturer’s instructions, from healthy subject PBMCs. IgD^+^ B cells were isolated from human tonsils by positive selection using biotin-anti-human IgD mAb (clone IA6-2; 348212, Biolegend) and MagniSort™ Streptavidin Positive Selection Beads (MSPB-6003-74, Thermo Fisher Scientific). Naïve IgM^+^IgD^+^ B cells were stimulated with CD154 (10 U/ml) or CpG ODN 2395 (1.0 μM; Custom synthesized by Eurofins Genomics) plus nil, IL-2 (20 ng/ml; BioLegend), IL-4 (20 ng/ml; R&D Systems), IL-15 and/or IL-21 (50 ng/ml; R&D Systems). Human B cells were cultured in FBS-RPMI at 37 °C in 48-well plates for 24, 48, 72, 96 and 120 h.

### Flow cytometry

For surface staining, mouse mononuclear white cells were reacted with BV421-anti-CD19 mAb (clone 1D3, BD), PE-anti-IgM mAb (clone RMM-1, 406507, BioLegend), PerCP/Cyanine5.5-anti-IgM mAb (clone RMM-1, 406512, BioLegend), APC-anti-mouse IgD mAb (clone 11-26 c.2a, 405713, BioLegend), PE/Cyanine7 anti-mouse CD138 mAb (clone 281-2, 142513, BioLegend), or 7-AAD. Human mononuclear white cells were reacted with PE/Cyanine7 anti-human CD19 mAb (clone HIB19, 302216, BioLegend), APC/Fire™ 750-anti-human IgM mAb (clone MHM-88, 314545, BioLegend), BV421-anti-human IgD mAb (clone IA6-2, 562518, BD Horizon™), BV785™-anti-human IgD mAb (clone IA6-2, 348241, BioLegend), PE-anti-human CD27 mAb (clone M-T271, 356405, BioLegend), or BV650™-anti-human CD38 mAb (clone HB-7, 356619, BioLegend).

For intracellular staining, mouse and human cells were pre-stained for select surface markers and treated with Fixable Viability Dye eFluor™ 780 (Fisher Scientific) and then incubated with the BD Cytofix/Cytoperm buffer at 4 °C for 45 min. After washing twice with the BD Perm/Wash buffer, cells were resuspended in HBSS with 1% BSA and stored overnight at 4 °C. Mouse cells were then stained with FITC-anti-Zfp318 Ab (ARP32523 P050, Aviva Systems Biology; labeled with FITC using iLink™ Antibody Labeling Kits, ABP Biosciences), FITC-anti-mouse IgD mAb (clone 11-26 c.2a, 405703, BioLegend) or PE-anti-mouse Blimp-1 mAb (clone 5E7, 150005, BioLegend). Human cells were then stained with FITC-anti-Zfp318 Ab, Alexa Fluor^®^ 647-anti-human IgD mAb (clone IA6-3, 348227, BioLegend) or Alexa Fluor^®^ 488-anti-human BLIMP1 mAb (clone 646702, IC36081G, R&D Systems). FACS analysis was performed on single cell suspensions. In all flow cytometry experiments, cells were appropriately gated on forward and side scattering to exclude dead cells and debris. Cell analyses were performed using a LSR-II or Celesta flow cytometer (BD Biosciences), and data were analyzed using FlowJo software (TreeStar)^67^. All experiments were performed in triplicates.

### Fluorescence microscopy

To analyze IgM and IgD-producing cells in the lamina propria and PPs, the intestine was folded into a “Swiss-roll”, fixed with PFA (4%) and embedded in paraffin. Ten-micrometer sections were cut and heated at 80 °C to adhere to the slide, washed four times in xylene for 2 min, dehydrated two times with 100% ethanol for 1 min, two times with 95% ethanol for 1 min, and washed two times in water for 1 min. Antigens were unmasked using 2 mM EDTA in 100 °C for 40 mins followed by a cooling step at 25 °C on the bench top, three times washing with 1x TBS and blocking using 10% BSA for 15 min. Slides were again washed three times with 1x TBS and stained with FITC-anti-IgD mAb (clone 11-26 c.2a, 405713, BioLegend), PE anti-mouse IgM mAb (clone II/41, 12-5790-82, Invitrogen), and FITC-anti-mouse IgA mAb (clone mA-6E1, 11-4204, Invitrogen) for 2 h in a dark moist chamber. After washing three times with Triton X-100 (0.1%) in TBS, slides were air-dried, and coverslips were mounted with ProLong^®^ Gold Antifade Reagent with DAPI (Invitrogen). Fluorescence images were captured using a 10x objective lens with a Zeiss Axio Imager Z1 fluorescence microscope. To analyze IgD-producing cells in MLNs, 10 μm MLN sections were prepared by cryostat and loaded onto positively charged slides, fixed in cold acetone, and stained with FITC-anti-IgD mAb (clone 11-26 c.2a, 405704, BioLegend), or PE anti-mouse IgM mAb (clone RMM-1, 406507, BioLegend), respectively, for 1 h at 25 °C in a moist chamber. Coverslips were then mounted using ProLong^®^ Gold Antifade Reagent using DAPI (Thermo Fisher), before examination with a fluorescence microscope.

### Mouse and human antibodies and autoantibodies

Titers of mouse serum, BALF or fecal total IgM, IgG1 and IgA and OVA-binding IgD, IgM, IgG1 and IgA, mouse serum dsDNA- or histone-binding IgD and total IgD in in vitro culture supernatants of stimulated human B cells, as well as human serum total and dsDNA-, RNA-, histone- or RNP/Sm-binding IgD were measured using specific ELISAs, as we described^20,25,68,69^. Total IgD in in vitro culture supernatants of stimulated mouse B cells or in mouse serum, BALF or feces were measured by dot-blotting with serially two-fold diluted samples. Mouse and human antinuclear autoantibodies (ANAs) were detected using Hep-2 cells and specific FITC-anti-mouse IgD (clone 11-26 c.2a, 405704, BioLegend) and FITC-anti-human IgD (clone IA6-2, 348205, BioLegend).

Bacteria-bound IgD and IgA were detected in feces by flow cytometry, as we described^27^. Briefly, feces (10 mg) were suspended in 100 μl 1x PBS (filtered through 0.2 μm filter), homogenized and centrifuged at 400 × *g* for 5 min to remove large particles. The supernatant was then centrifuged at 8000 g for 10 min to remove non-bound antibodies. The bacterial pellet was suspended in 1 ml of PBS with 1% (w/v) BSA. After fixation with 7.2% formaldehyde for 10 min at room temperature, bacteria were washed with PBS, and stained with PE-anti-IgD mAb (clone 11-26 c.2a; 405705, BioLegend) or PE-anti-mouse IgA mAb (clone mA-6E1, 12-4204-82, Invitrogen) on ice for 30 min, washed with PBS, and further resuspended in 1 × PBS containing 0.2 μg ml^−1^ DAPI for flow cytometry analysis. All events that stained with DAPI were considered as bacteria.

### S–S region DNA recombinations and S region somatic mutations

Genomic DNA was prepared from human or mouse B cells using QIAmp DNA Mini Kit (Qiagen) or from paraffin-embedded human IgD or IgA myeloma tissue sections (obtained from the University of Arkansas for Medical Science) using Quick-DNA™ FFPE Kit (Zymo Research). Recombined Sμ–σδ, Sμ–Sγ1, Sμ–Sα, and Sμ–Sε DNA were amplified by specific nested PCR involving two sequential rounds of PCR using Phusion™ high-fidelity DNA polymerase (Thermo Scientific™) and two sets of nested specific primers (forward Iμ and reverse Cδ or Sx primers) (Custom synthesized by Eurofins Genomics, Supplementary Table 1). The first and second rounds of PCR were performed at 98 °C for 30 sec, 58 °C for 45 sec, 72 °C for 4 min (30 cycles). Amplified genomic DNA was fractionated through 1.0% agarose, blotted onto Hybond-N^+^ membranes (GE Healthcare) and hybridized to biotin-labeled internal Sμ and σδ, Sγ1, Sγ3, Sα, or Sε specific probes^70^. Detection was performed using the Chemiluminescent Nucleic Acid Detection Module (Thermo Fisher Scientific) according to the manufacturer’s instructions. For sequence analysis of the recombined DNA, PCR products were purified using a QIAquick PCR purification kit (Qiagen). The amplified library was tagged with barcodes for sample multiplexing, and PCR was enriched and annealed to the required Illumina clustering adapters. High-throughput 300–base pair (bp) paired-end sequencing was performed by the University of Texas Health Science Center San Antonio Genome Sequencing Facility using the Illumina MiSeq platform. S–S junctions and somatic mutations in the S regions were analyzed by sequence alignment as performed by comparing PCR products sequences with germline Sμ and σσδ, Sγ1, or Sα sequences using National Center for Biotechnology Information BLAST (www.ncbi.nih.gov/BLAST).

### RT-PCR and quantitative RT-PCR (qRT-PCR)

For quantification of mRNA, germline *I*_*H*_−*C*_*H*_, post-recombination *Iμ-C*_*H*_ and mature *V*_*H*_*DJ*_*H*_−*C*_*H*_ transcripts, RNA was extracted from 0.2–5.0 × 10^6^ cells using either Trizol^®^ Reagent (Invitrogen) or RNeasy Plus Mini Kit (Qiagen). Residual DNA was removed from the extracted RNA with gDNA eliminator columns (Qiagen). cDNA was synthesized from total RNA with the SuperScript™ IV First-Strand Synthesis System (Thermo Fisher) using oligo-dT primer. Transcript expression was measured by qRT-PCR with the appropriate primers (Supplementary Table 1) using a Bio-Rad MyiQ™ Real-Time PCR Detection System (Bio-Rad Laboratories) to measure SYBR Green (IQ™ SYBR^®^ Green Supermix, Bio-Rad Laboratories) incorporation with the following protocol: 95 °C for 15 sec, 40 cycles of 94 °C for 10 sec, 60 °C for 30 sec, 72 °C for 30 sec. Data acquisition was performed during 72 °C extension step. Melting curve analysis was performed from 72 °C–95 °C. Mature *V*_*H*_*DJ*_*H*_*−Cμm*, *V*_*H*_*DJ*_*H*_*−Cμs*, *V*_*H*_*DJ*_*H*_*−Cδm* and *V*_*H*_*DJ*_*H*_*−Cδs* transcripts were analyzed by semi-quantitative PCR using serially two-fold diluted cDNA.

### Western blotting

B cells were lysed in Laemmli buffer. Cell extracts containing equal amounts of protein (50-100 μg) were fractionated through SDS-PAGE (6%). The fractionated proteins were transferred onto polyvinylidene difluoride membranes (Bio-Rad) overnight (30 V/90 mA) at 4 °C. After blocking and overnight incubation at 4 °C with anti-AID Ab (H-80, Santa Cruz), anti-Ku70 Ab (A0883, Abclonal), anti-Ku86 Ab (A5862, Abclonal), anti-Rad52 Ab (H-300, Santa Cruz Biotechnology), anti-phospho-Rad52 Ab (Y408472, Applied Biological Materials Inc.) or anti-β-Actin mAb (clone 2F1-1, 643802, BioLegend), the membranes were incubated with appropriate horseradish peroxidase (HRP)-conjugated secondary antibodies. After washing with TBS-Tween 20 (0.05%), bound HRP-conjugated antibodies were detected using Western Lightning Plus-ECL reagents (PerkinElmer Life and Analytical Sciences).

### ChIP and qPCR

ChIP assays were performed as previously described^71–73^. Human or mouse B cells (1.0 × 10^7^) were treated with formaldehyde (1% v/v) for 10 min at 25 °C to crosslink chromatin, washed once in cold PBS with protease inhibitors (Roche) and resuspended in lysis buffer (20 mM Tris-HCl, 200 mM NaCl, 2 mM EDTA, 0.1% w/v SDS and protease inhibitors, pH 8.0). Chromatin was fragmented by sonication (DNA fragments of about 200–1000 bp in length), pre-cleared with protein A agarose beads (Pierce) and incubated with agarose conjugated anti-Rad52 mAb (clone F-7; sc-365341 AC, Santa Cruz Biotechnology, 5 μg ml^−1^), anti-Ku70/86 mAb (MA1-21818, Thermo Fisher Scientific, 5 μg ml^−1^), or control rabbit or mouse IgG mAb with irrelevant specificity at 4 °C overnight. Immune complexes were directly precipitated (samples incubated with the agarose conjugated anti-Rad52 mAb), or precipitated by Protein A agarose beads, then washed and eluted (50 mM Tris-HCl, 0.5% SDS, 200 mM NaCl, 100 μg/ml proteinase K, pH 8.0), followed by incubation at 65 °C for 4 h. DNA was purified using a QIAquick PCR purification kit (Qiagen). The Sμ or σδ region DNA was amplified from immunoprecipitated chromatin by qPCR using appropriate primers (Custom synthesized by Eurofins Genomics, Supplementary Table 1). Data were normalized to input chromatin DNA and depicted as relative abundance of each amplicon.

### *RAD52* knockdown in human B cells

The human RAD52-specific siRNA oligo duplex (TT320001, Locus ID 5893) and non-effective Trilencer-27 Flurescence-labeled transfection control siRNA duplex (SR30002) were obtained from Origene Technologies. The siRNA duplexes were used to transfect purified human naïve B cells using the Human B Cell Nucleofector^TM^ Kit (VPA-1001, LONZA) and Amaxa Nucleofector^TM^ Device (LONZA). Transfected B cells were then stimulated with CpG ODN 2395 (Custom synthesized by Eurofins Genomics) plus IL-2 and IL-21 for 96 h before genomic DNA extraction for analysis of Sμ–σδ and Sμ–Sγ1 DNA recombination. Expression of *RAD52* and *AICDA* transcripts were analyzed by qRT-PCR using specific primers 24 h after transfection. Expression of RAD52, AID, and β-ACTIN proteins were analyzed by immune-blotting 24 h after transfection.

### High-throughput mRNA-Seq

RNA was isolated from cells using the Directzol RNA Microprep Kit (Zymogen Research), according to manufacturer’s instructions and as previously described^69^. RNA integrity was verified using an Agilent Bioanalyzer 2100 (Agilent). Next generation RNA-Seq for mRNA and non-coding RNA was performed by the Genome Sequencing Facility at University of Texas Health Science Center San Antonio. High-quality RNA was processed using an Illumina TruSeq RNA sample prep kit v2 or TruSeq Small RNA Sample Prep kit following the manufacturer’s instructions (Illumina). Clusters were generated using TruSeq Single-Read Cluster Gen. Kit v3-cBot-HS on an Illumina cBot Cluster Generation Station. After quality control procedures, individual mRNA-Seq or small RNA-Seq libraries were then pooled based on their respective 6-bp index portion of the TruSeq adapters and sequenced at 50 bp/sequence using an Illumina HiSeq 3000 sequencer. Resulting reads were checked by assurance (QA) pipeline and initial genome alignment (Alignment). After the sequencing run, demultiplexing with CASAVA was employed to generate the Fastq file for each sample. All sequencing reads were aligned with their reference genome (UCSC mouse genome build mm9) using TopHat2 default settings, and the Bam files from alignment were processed using HTSeq-count to obtain the counts per gene in all samples^67^. Quality control statistical analysis of outliers, intergroup variability and distribution levels, were performed for statistical validation of the experimental data.

### Statistical analysis

Statistical analysis was performed using Excel (Microsoft) or GraphPad Prism^®^ software. *P*-values were determined by paired and unpaired Student’s *t-*tests; *P*-values < 0.05 were considered significant.

### IRB for use of human tissues and peripheral blood as well as IACUC for use of mice

For the use of DNA procured from formalin-fixed paraffin-embedded tissues obtained from the University of Arkansas for Medical Science, the study was reviewed by the University of Arkansas for Medical Sciences Institutional Review Board (IRB) which determined that this project is not human subject research as defined in 45 CFR 46.102. Human B cells were purified from PBMCs of healthy subject buffy coats obtained from South Texas Blood and Tissue Center, San Antonio, Texas, under the “Healthy Volunteer Blood Donor Program”. SLE patient B cells were purified from PBMCs obtained under the University of Texas Long School of Medicine IRB HSC 20140234H (including informed consent) “Class switching, somatic hypermutation and plasma cell differentiation in B cells”. Informed consents were obtained from the study participants. Studies involving mice and mouse-derived materials were performed under the University of Texas Long School of Medicine IACUC 20200019AR “Somatic hypermutation, class-switch DNA recombination and plasma cell differentiation in antibody and autoantibody responses”.

### Antibodies

All polyclonal Abs and mAbs used in these studies are listed in Supplementary Table 2, including Cat. number, clone number (for mAbs), vendor and working dilution.

### Reporting summary

Further information on research design is available in the Nature Research Reporting Summary linked to this article.

## Supplementary information

Supplementary Information

Reporting Summary

## Data Availability

The mRNA-sequencing data are available in NCBI Gene Expression Omnibus (GEO) through GEO Series accession number GSE156904. S–S junction sequencing data has been deposited in NCBI Sequence Read Archive (SRA) under BioProject PRJNA789895. The raw numbers for charts and graphs are available in the Source Data file whenever possible. The source data underpinning Figs. 2a–c, 3a, c, 4a, 5a–f, 6a–f, 7a, c–h, j, k, 8a–e, 9c, and 10a–c, g–k are available in the Source Data file. All other data supporting the findings of this study are available within the article and its supplementary information files and from the corresponding author upon reasonable request. Source data are provided with this paper.

## Source data

Source Data
