# Supplementary material for: Rad52 mediates class-switch DNA recombination to IgD
Source: Nat Commun. 2022 Feb 21;13:980. doi: 10.1038/s41467-022-28576-2 (PMC8861003; doi:10.1038/s41467-022-28576-2)
Supplement: Supplementary file 1 — Supplementary Information [file 41467_2022_28576_MOESM1_ESM.pdf]

SUPPLEMENTARY MATERIAL

Rad52 mediates class-switch DNA recombination to IgD

Yijang Xu, Hang Zhou, Ginell Post, Hong Zan and Paolo Casali

Supplementary Figure 1. *In vivo* human B cell intra–σδ deletional recombination junctions and inter Sμ-σδ DNA switch recombination junctions contain high frequencies of microhomologies.

Supplementary Figure 2. *In vivo* mouse B cell intra–σδ deletional recombination junctions and inter Sμ-σδ DNA switch recombination junctions contain high frequencies of microhomologies.

Supplementary Figure 3. Mouse B cells stimulated to undergo CSR to IgD and IgG1 *in vitro* display a higher frequency of microhomologies in Sμ-σδ DNA recombination junctions.

Supplementary Figure 4. Human B cells stimulated to undergo CSR to IgD and IgG1 *in vitro* display a higher frequency of microhomologies in Sμ-σδ DNA recombination junctions.

Supplementary Figure 5. *Rad52*$^{-/-}$ mice B cells display no Sμ–σδ recombination and even lower frequencies of microhomologies in Sμ-Sγ1 junctions.

Supplementary Figure 6. *Rad52*$^{-/-}$ mouse B cells display lower frequencies of microhomologies in Sμ-Sα junctions.

Supplementary Figure 7. Gating strategy for flow cytometry analysis.

Supplementary Figure 8. Stimuli that induce IgD CSR downregulate Zfp318 in B cells.

Supplementary Figure 9. Gating strategy for flow cytometry analysis.

Supplementary Figure 10. Increased B cell CSR to IgD in mice with systemic autoimmunity involves a high frequency of microhomologies in recombined Sμ-σδ, Sμ-Sγ1 and Sμ-Sα DNA junctions.

Supplementary Figure 11. Rad52 mediates Sμ-σδ DNA recombination (CSR to IgD).

Supplementary Table 1. Oligonucleotide primers used in these studies.

Supplementary Table 2. Antibodies used in these studies.

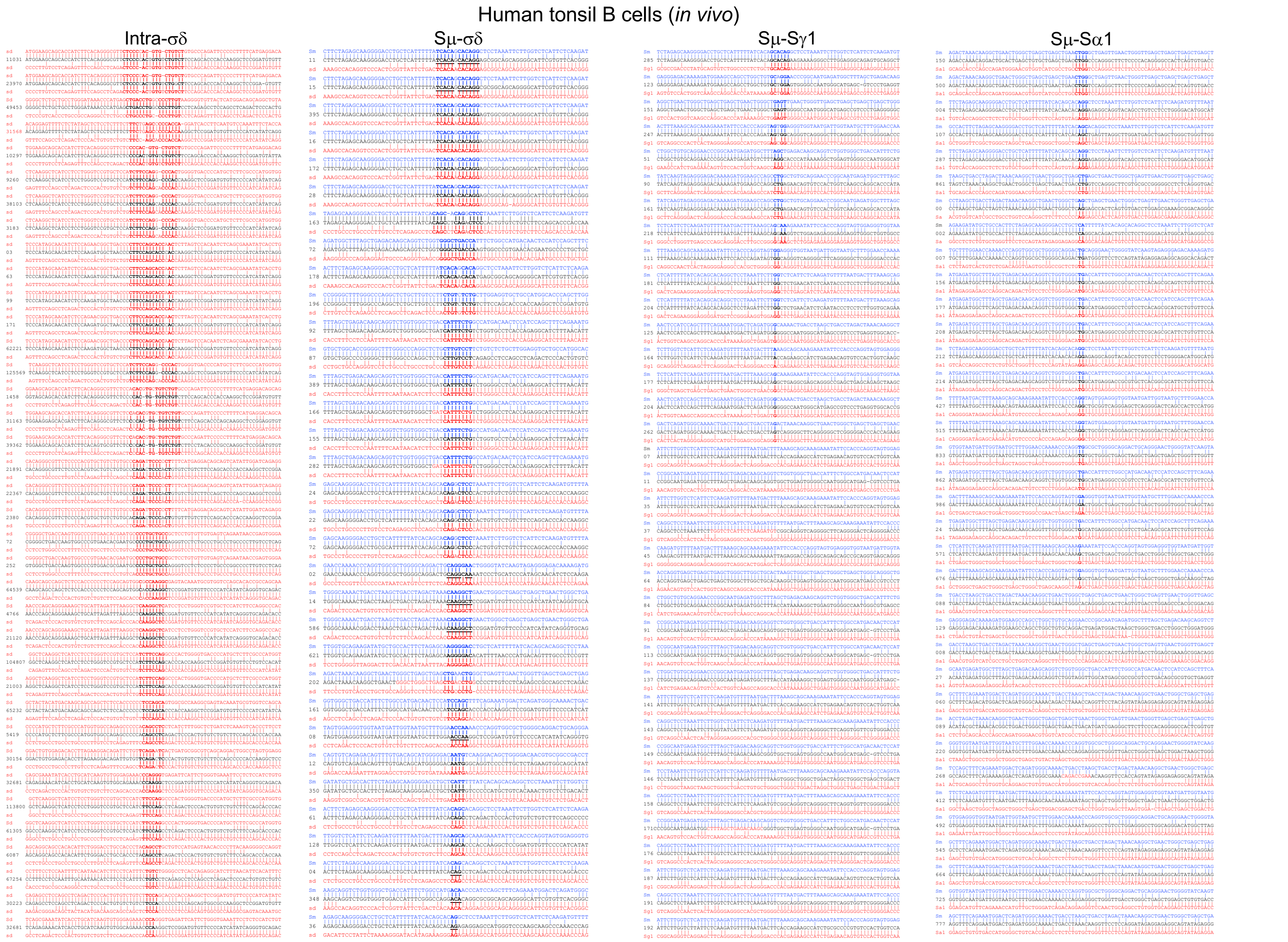

Supplementary Fig. 1 | *In vivo* human B cell intra–σδ deletional recombination junctions and inter Sμ-σδ DNA switch recombination junctions contain high frequencies of microhomologies. Amplified intra–σδ junctional DNAs and inter Sμ-σδ, Sμ-Sγ1 and Sμ-Sα1 junctional DNAs from human tonsil B cells were amplified and sequenced using MiSeq system. Thirty-two representative intra-σδ, inter Sμ-σδ, Sμ-Sγ1 or Sμ-Sα1 junctional sequences are shown in each column. Each sequence is compared with the corresponding germline σδ (above, red) or Sμ (above, blue) and σδ, Sγ1 or Sα1 (below, red) sequences. Microhomologies (bold) were determined by identifying the longest region at the σδ–σδ, Sμ-σδ, Sμ-Sγ1 or Sμ-Sα1 junction of perfect uninterrupted donor/acceptor identity or the longest overlap region at the S–S junction with no more than one mismatch on either side of the breakpoint.

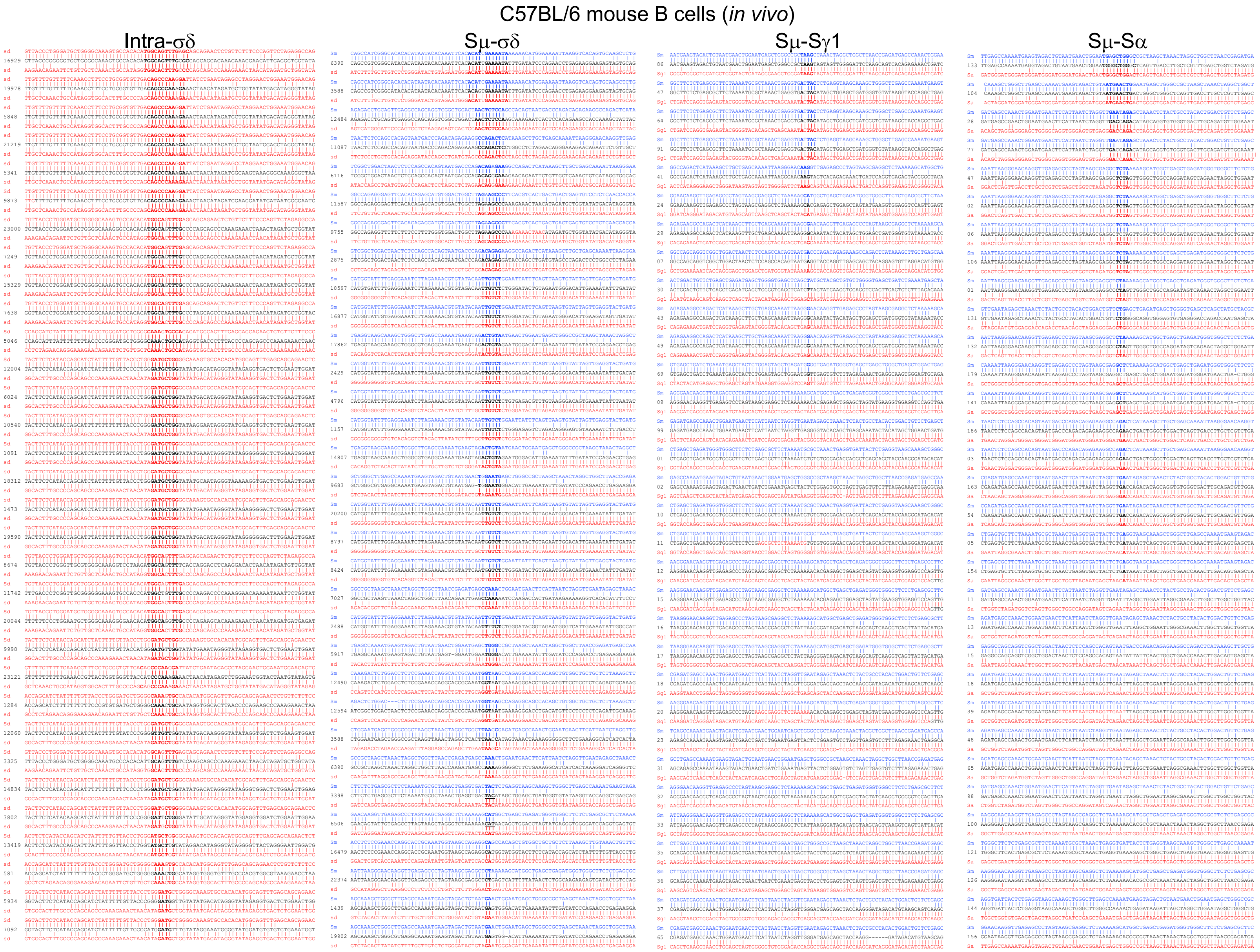

Supplementary Fig. 2 | *In vivo* mouse B cell intra–σδ deletional recombination junctions and inter Sμ-σδ DNA switch recombination junctions contain high frequencies of microhomologies. Amplified intra–σδ junctional DNAs and inter Sμ-σδ, Sμ-Sγ1 and Sμ-Sα1 junctional DNAs from spleen B cells of an OVA-immunized C57BL/6 mouse were amplified and sequenced using MiSeq system. Thirty-two representative intra-σδ, inter Sμ-σδ, Sμ-Sγ1 or Sμ-Sα1 junctional sequences are shown in each column. Each sequence is compared with the corresponding germline σδ (above, red) or Sμ (above, blue) and σδ, Sγ1 or Sα1 (below, red) sequences. Microhomologies (bold) were determined by identifying the longest region at the σδ–σδ, Sμ-σδ, Sμ-Sγ1 or Sμ-Sα1 junction of perfect uninterrupted donor/acceptor identity or the longest overlap region at the S–S junction with no more than one mismatch on either side of the breakpoint.

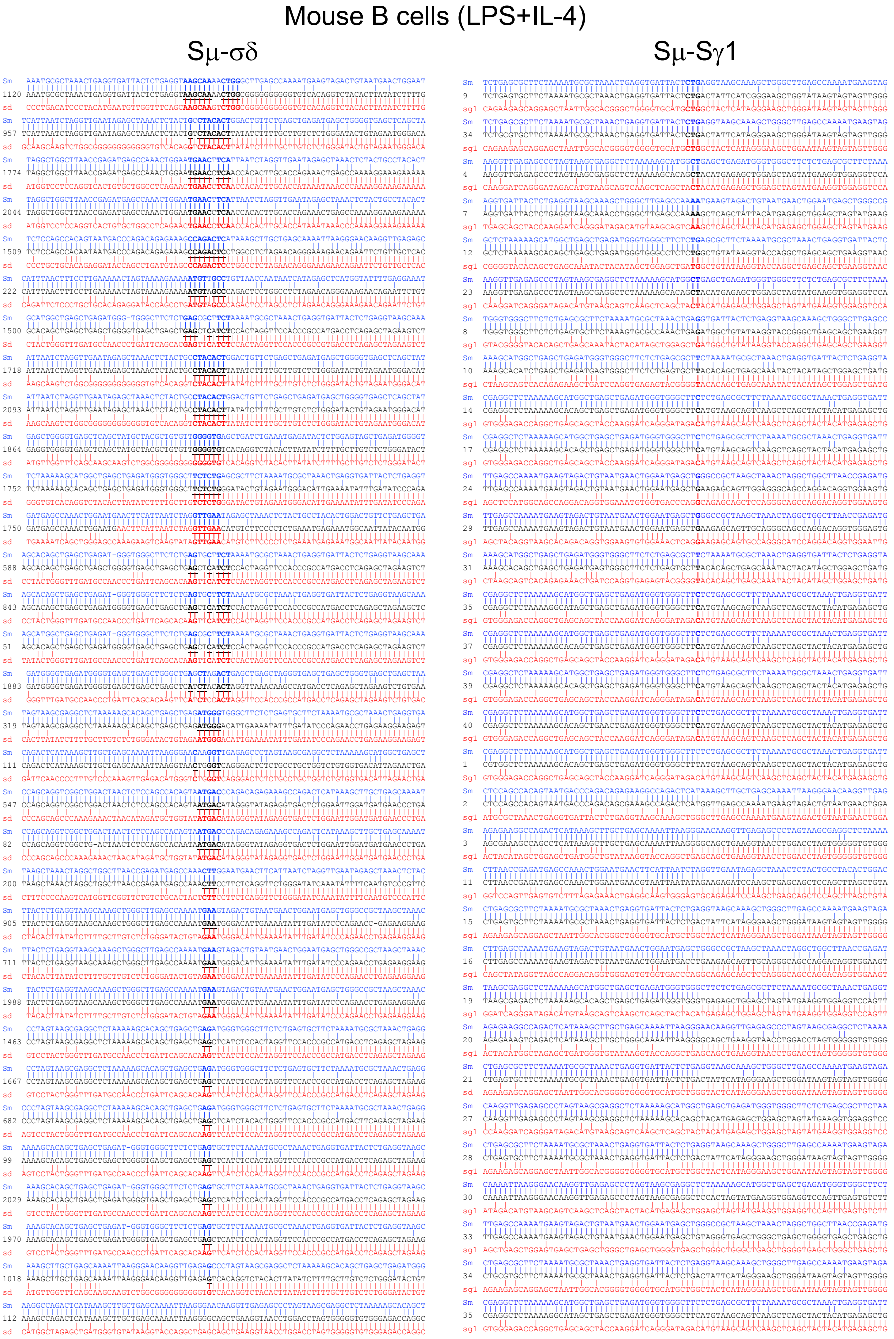

Supplementary Fig. 3 | Mouse B cells stimulated to undergo CSR to IgD and IgG1 *in vitro* display a higher frequency of microhomologies in Sμ-σδ DNA recombination junctions. C57BL/6 mouse B cells were stimulated with LPS plus IL-4 and cultured for 96 h – these stimuli induce CSR to IgD and IgG1. Junctions of recombined Sμ–σδ and Sμ-Sγ1 DNAs were amplified and sequenced using MiSeq system. Thirty-two representative Sμ-σδ and 32 representative Sμ-Sγ1 junctional sequences are shown. Each recombinant DNA sequence (middle) is compared with the germline Sμ (above, blue) and the σδ or Sγ1 (below, red) sequence. Microhomologies (bold) were determined by identifying the longest region at the Sμ-σδ or Sμ-Sγ1 junction of perfect uninterrupted donor/acceptor identity or the longest overlap region at the S–S junction with no more than one mismatch on either side of the breakpoint.

Human IgM+IgD+ B cells induced to undergo CSR to IgD and IgG1 by CpG+IL-2+IL-21

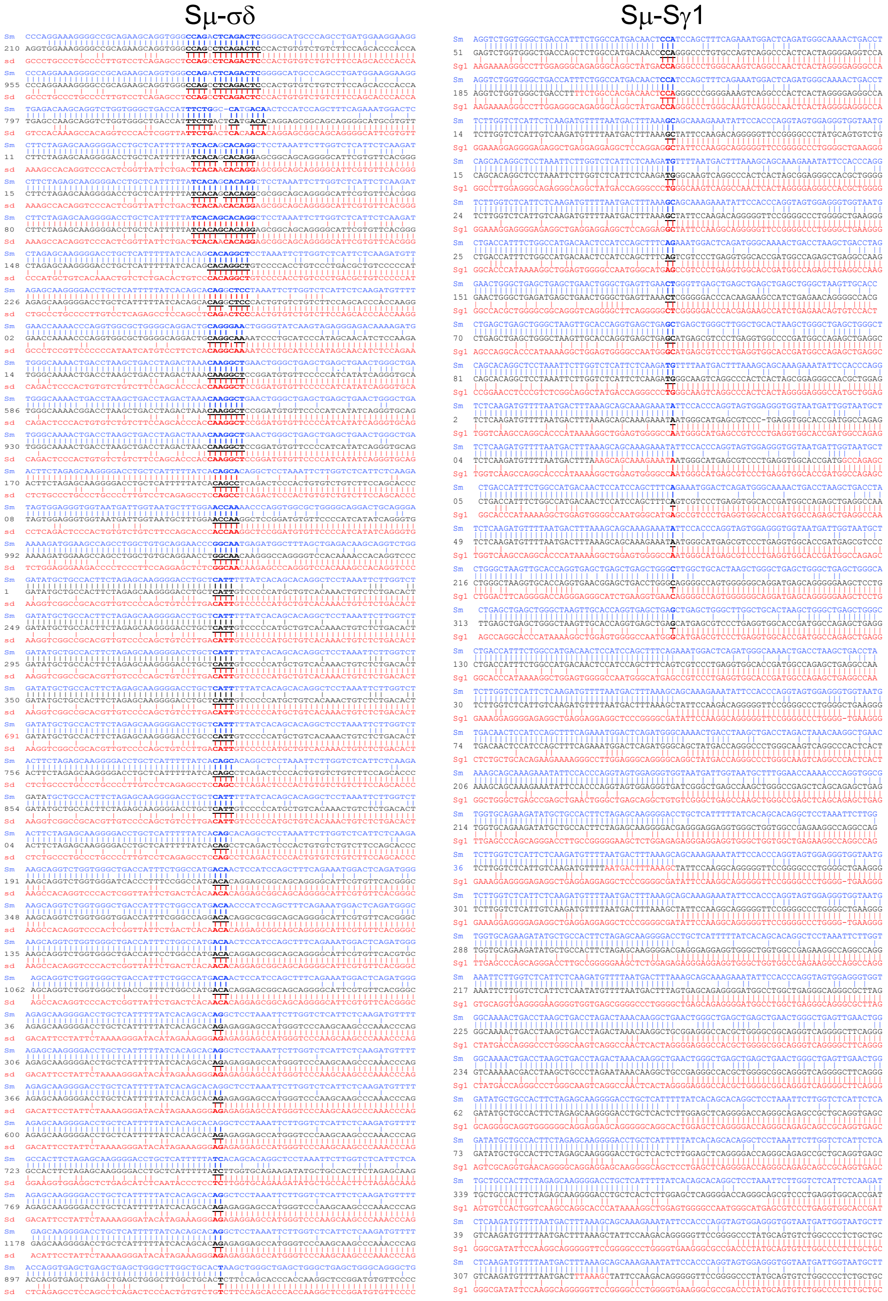

Supplementary Fig. 4 | Human B cells stimulated to undergo CSR to IgD and IgG1 *in vitro* display a higher frequency of microhomologies in Sμ-σδ DNA recombination junctions. Human naïve B cells were stimulated with CpG plus IL-2 and IL-21 and cultured for 120 h. Junctions of recombined Sμ-σδ and Sμ-Sγ1 DNAs were amplified and sequenced using MiSeq system. Thirty-two representative junctional Sμ-σδ sequences and 32 representative Sμ-Sγ1 sequences are shown. Each recombinant DNA sequence (middle) is compared with germline Sμ (above, blue) and σδ or Sγ1 (below, red) sequences. Microhomologies (bold) were determined by identifying the longest region at the Sμ-σδ or Sμ-Sγ1 junction of perfect uninterrupted donor/acceptor identity or the longest overlap region at the S-S junction with no more than one mismatch on either side of the breakpoint.

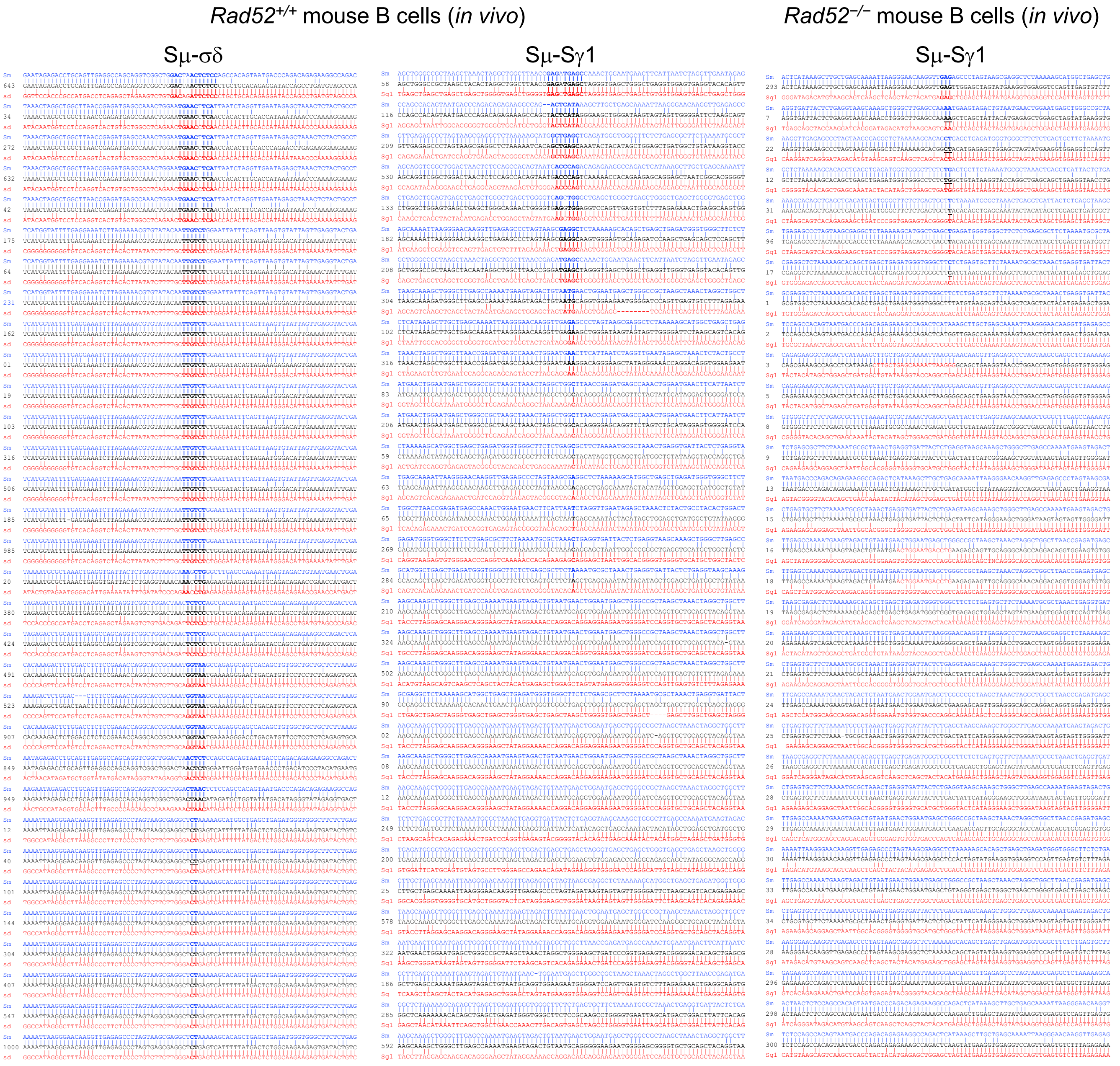

Supplementary Fig. 5 | *Rad52*–/– mice B cells display no Sμ–σδ recombination and even lower frequencies of microhomologies in Sμ-Sγ1 junctions. Junctions of recombined Sμ–σδ and Sμ-Sγ1 DNAs from spleen B cells of OVA-immunized *Rad52*+/+ and *Rad52*–/– mice were amplified and sequenced using MiSeq system. No Sμ–σδ recombination was detected in B cells from *Rad52*–/– mice. Thirty-two representative Sμ-σδ and 32 representative Sμ-Sγ1 junctional sequences are shown. Each recombinant DNA sequence (middle) is compared with germline Sμ (above, blue) and σδ or Sγ1 (below, red) sequences. Microhomologies (bold) were determined by identifying the longest region at the Sμ-σδ or Sμ-Sγ1 junction of perfect uninterrupted donor/acceptor identity or the longest overlap region at the S-S junction with no more than one mismatch on either side of the breakpoint.

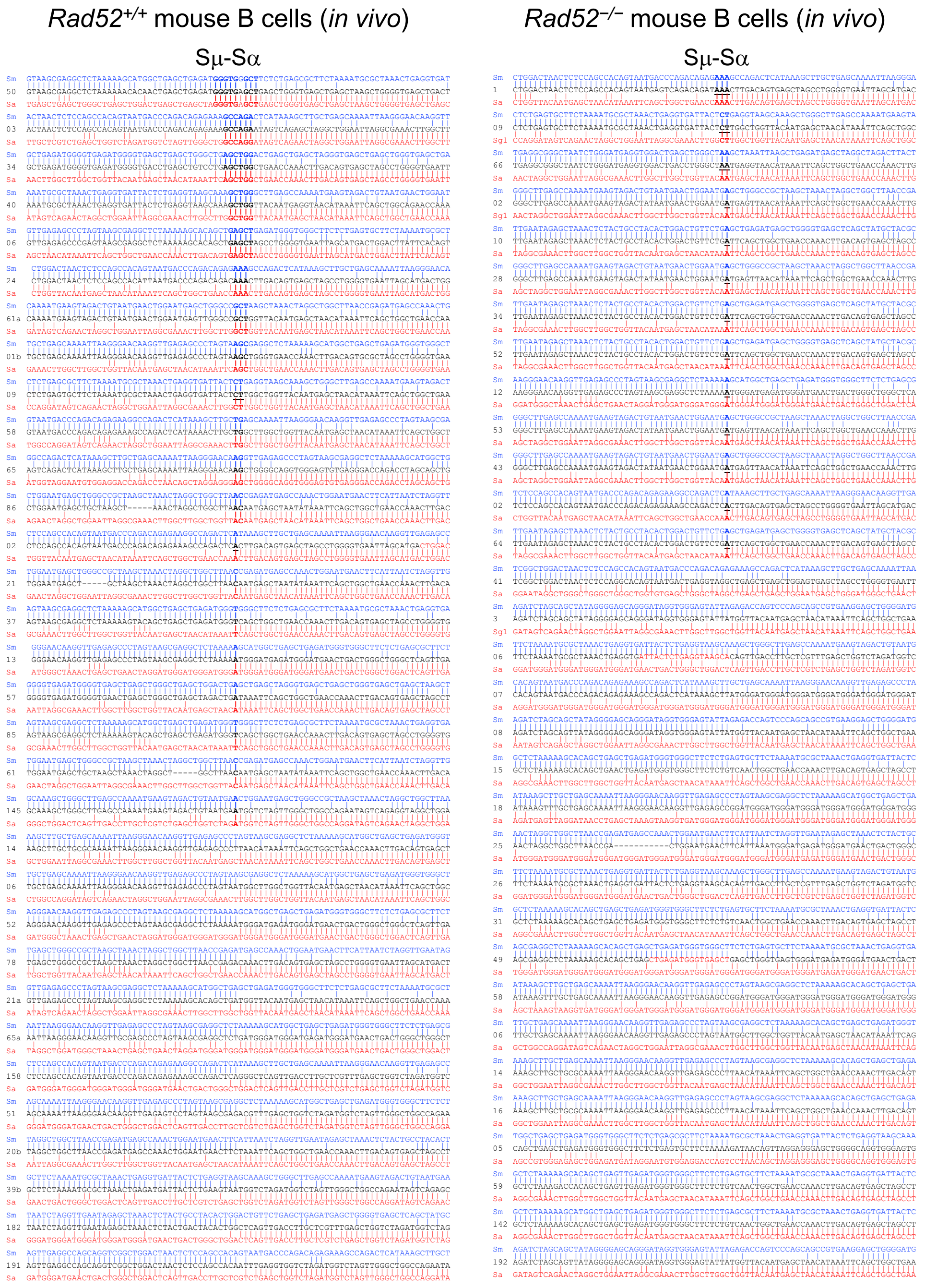

Supplementary Fig. 6 | *Rad52*$^{-/-}$ mouse B cells display lower frequencies of microhomologies in Sμ-Sα junctions. Junctions of recombined Sμ-Sα DNAs from spleen B cells of OVA-immunized *Rad52*$^{+/+}$ and *Rad52*$^{-/-}$ mice were amplified and sequenced using MiSeq system. Thirty-two representative junctional sequences from B cells of *Rad52*$^{+/+}$ mice and 32 representative sequences from B cells of *Rad52*$^{-/-}$ mice are shown. Each recombinant DNA sequence (middle) is compared with germline Sμ (above, blue) and Sα (below, red) sequences. Microhomologies (bold) were determined by identifying the longest region at the Sμ-Sα junction of perfect uninterrupted donor/acceptor identity or the longest overlap region at the S-S junction with no more than one mismatch on either side of the breakpoint.

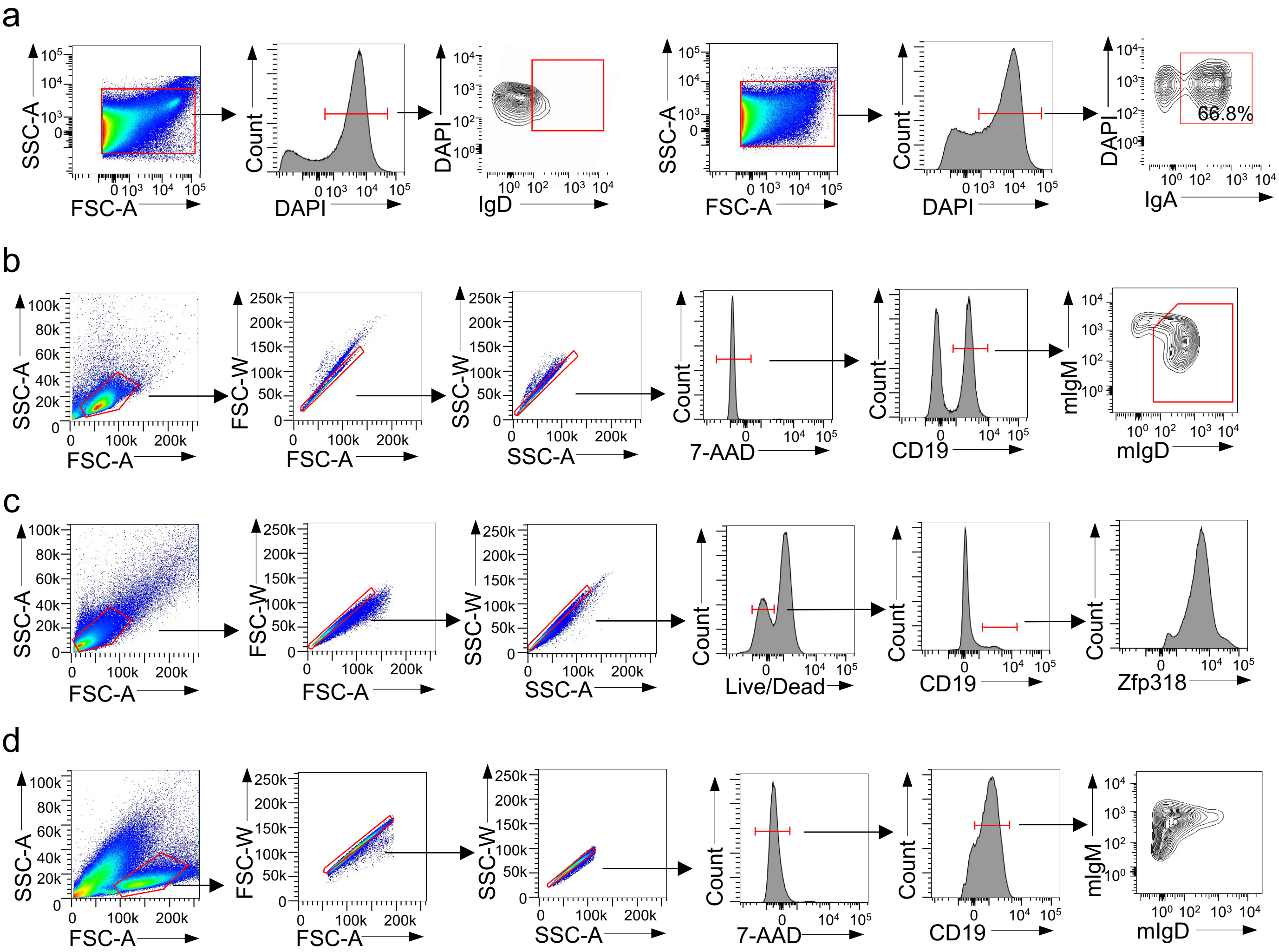

Supplementary Fig. 7 | Gating strategy for flow cytometry analysis. a, Gating strategy to analyze fecal bacteria-bound IgD and IgA presented in Fig. 5g. **b**. Gating strategy to analyze surface expression of IgM and IgD presented in Fig. 7a. **c**, Gating strategy to analyze intracellular Zfp318 protein level presented in Fig. 7e. **d**, Gating strategy to analyze surface expression of IgM presented in Fig. 7i.

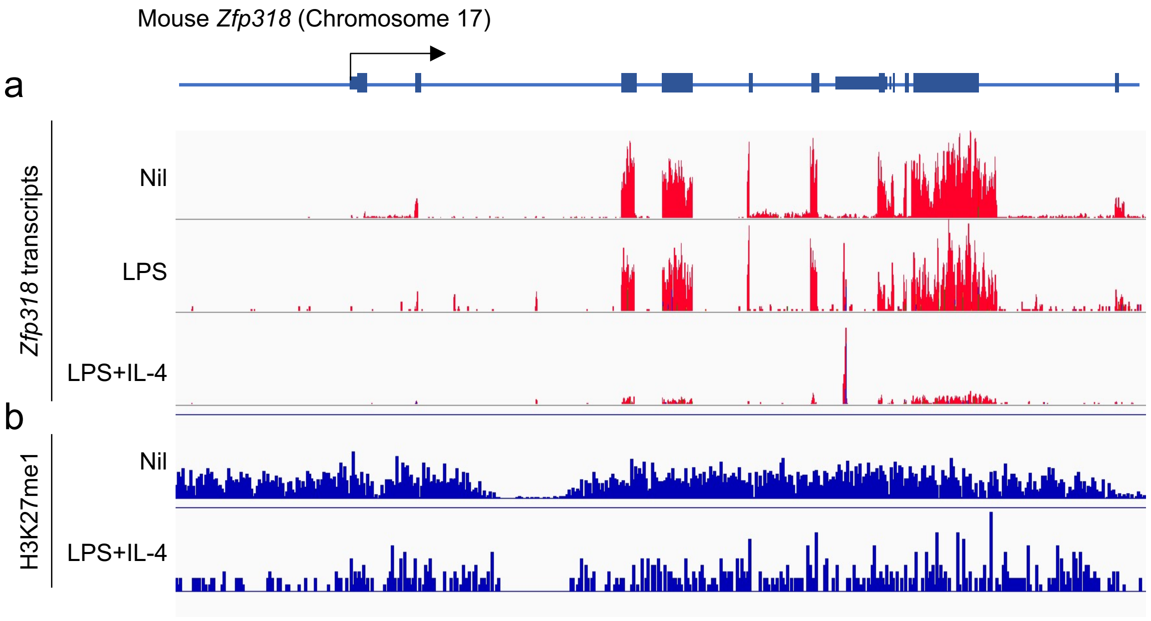

Supplementary Fig. 8 | Stimuli that induce IgD CSR downregulate Zfp318 in B cells. **a,** Expression of *Zfp318* transcripts in unstimulated mouse naïve B cells and mouse naïve B cells stimulated by LPS or LPS plus IL-4 for 72 h, as analyzed by mRNA-Seq. **b,** Histone H3K27me1, an activation mark, at mouse *Zfp318* locus, in resting B cells or B cells stimulated by LPS plus IL-4 for 48 h, as derived by elaboration of ChIP-Seq data by Kieffer-Kwon et al. (GSE82144, Vian et al, *Cell* 173:1165-1178, 2018).

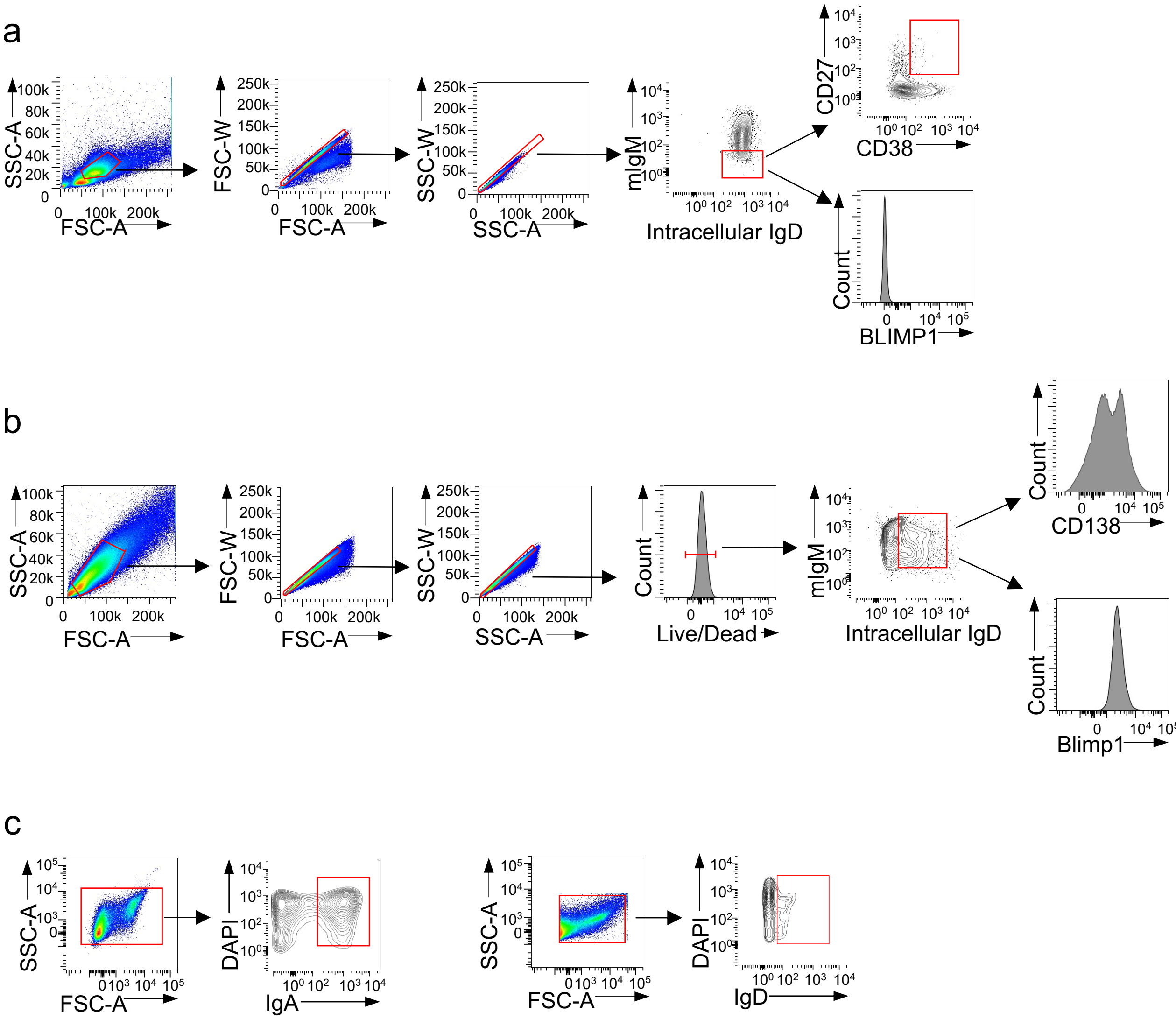

Supplementary Fig. 9 | Gating strategy for flow cytometry analysis. a, Gating strategy to analyze surface IgM, and intracellular IgD and BLIMP-1 presented in Fig. 9a. **b**. Gating strategy to analyze surface IgM and CD138, and intracellular IgD and Blimp-1 presented in Fig. 9b. **c**, Gating strategy to analyze fecal bacteria-bound IgD and IgA presented in Fig. 10e.

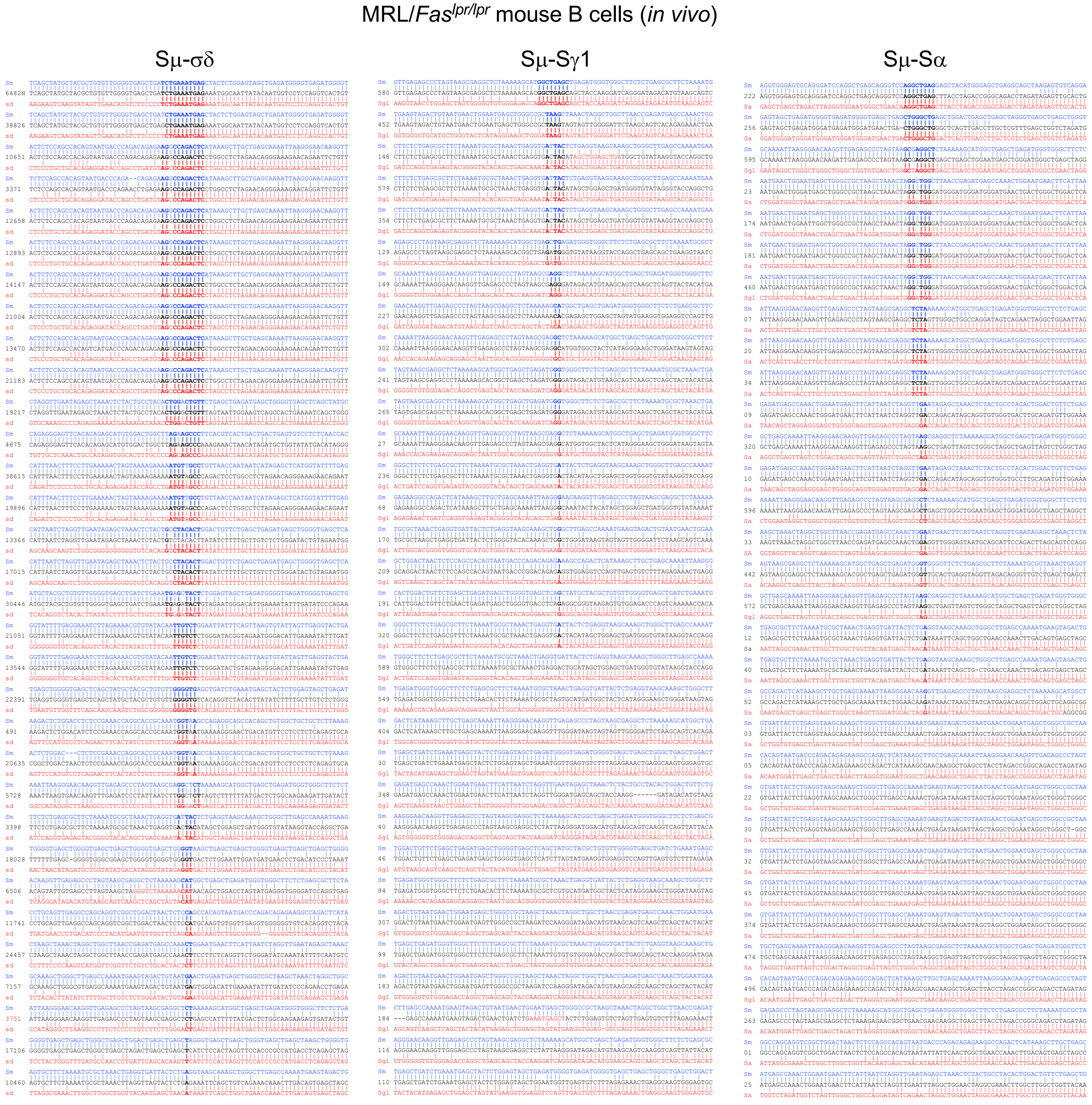

Supplementary Fig. 10 | Increased B cell CSR to IgD in mice with systemic autoimmunity involves a high frequency of microhomologies in recombined Sμ-σδ, Sμ-Sγ1 and Sμ-Sα DNA junctions. Junctions of Sμ-σδ, Sμ-Sγ1 and Sμ-Sα recombinant DNAs from spleen B cells of MRL/*Fas*$^{lpr/lpr}$ mice were amplified and sequenced by MiSeq. Thirty-two representative junctional Sμ-σδ, Sμ-Sγ1 and Sμ-Sα sequences are shown. Each recombinant DNA sequence (middle) is compared with the germline Sμ (above, blue) and σδ, Sγ1 or Sα (below, red) sequences. Microhomologies (bold) were determined by identifying the longest region at the Sμ-σδ, Sμ-Sγ1 or Sμ-Sα junction of perfect uninterrupted donor/acceptor identity or the longest overlap region at the S-S junction with no more than one mismatch on either side of the breakpoint.

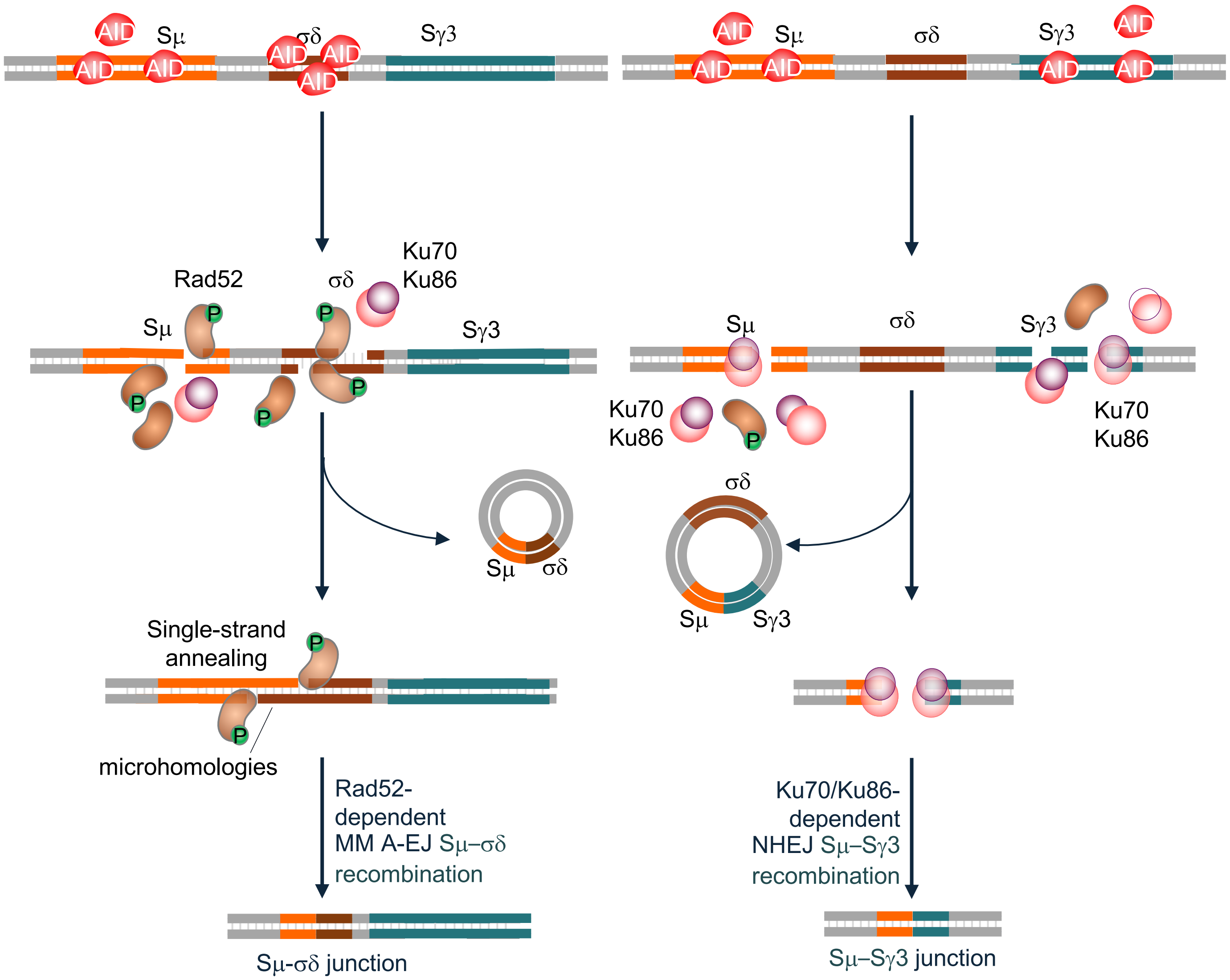

Supplementary Fig. 11 | Rad52 mediates Sμ-σδ DNA recombination (CSR to IgD). CSR is initiated by AID-mediated generation of multiple DSBs in the targeted upstream Sμ and downstream Sγ3 (shown here), Sγ1, Sγ2b, Sγ2a, Sε or Sα region. Ku70/Ku86, a core NHEJ factor, binds to upstream (Sμ) and downstream (Sγ3 , Sγ1, Sγ2b, Sγ2a, Sε or Sα) region blunt DSB ends to synapse them by NHEJ, leading to long-range inter-S–S region recombination and CSR to IgG3, IgG1, IgG2b, IgG2a, IgE or IgE. Rad52, an HR element, which is phosphorylated upon CSR induction, binds preferentially to single-strand overhangs of resected DSBs and facilitates in a (Ku70/Ku86-independent) microhomology-mediated (MM) A-EJ, which favors short-range intra-S region recombination, such as Sμ-Sμ or σδ–σδ, but can also efficiently mediate short-range inter-S–S region recombination, particularly Sμ-σδ recombination, leading to CSR to IgD. To this end, the B cell recruits the CSR machinery to transcribe Sμ and σδ regions and AID, which introduce DSBs into these two S regions. The Sμ and σδ region DSB ends undergo abundant resection, yielding single-strand DNA overhangs. Upstream Sμ and downstream σδ DSB complementary overhangs are rejoined by Rad52 through MM A-EJ, thereby completing CSR to IgD. In a physiological context, Rad52 also resolves by MM A-EJ a minor proportion of long-range Sμ–Sγ1, –Sγ2b, –Sγ2a, –Sε and –Sα recombination. In the absence of Ku70/Ku86 such as in *Ku70$^{-/-}$/Ku86$^{-/-}$* B cells, Rad52 mediates all ”residual” long-range Sμ–Sγ1, –Sγ2b, –Sγ2a, –Sε or –Sα recombination.

Supplementary Table 1 | Primers used for this study.

	Forward primer	Reverse primer
Human and mouse genes		
Human		
ZFP318	5’-CCTGGGGAATCTGGGGGATA-3’	5’-GCGGGATCGGAGGAATTACA-3’
RAD52	5’-GTAGGGAGAGGCTCTGGACA-3’	5’-GCAGGTGCTTAGGACCAAGT-3’
KU70	5’-GAAGCAAAAGGCCCAAGGTG-3’	5’-AGCAGCTCCTGCTTCTTCAG-3’
KU86	5’-GCAGTGTCACCTCTGTTGGA-3’	5’-GCTCGGATGCAGTCTATGCT-3’
AICDA	5’-GTCACCTGGTTCACCTCCTG-3’	5’-CTTGCGGTCCTCACAGAAGT-3’
β-ACTIN	5’-AGAGCTACGAGCTGCCTGAC-3’	5’-AGCACTGTGTTGGCGTACAG-3’
Mouse		
Zfp318	5’-CGTAGTCGTCCCAATCTCCG-3’	5’-TGGAATGGACACCCGAACAG-3’
Rad52	5’-CATTGGGACTCCCCAAACCA-3’	5’-GCGAGTCTCCATCTGTTCCC-3’
Ku70	5’-CACCAAGCGGTCTCTGACTT-3’	5’-AGAGAGGGCCTCAGGTAGTG-3’
Ku86	5’-AGGCCCAGGAAGCTCTATCA-3’	5’-GCACTCTTGGATTCCCCACA-3’
Aicda	5'-AGAAAGTCACGCTGGAGACC-3'	5'-CTCCTCTTCACCACGTAGCA-3'
β-Actin	5’-CTAAGGCCAACCGTGAAAG-3’	5’-ACCAGAGGCATACAGGGACA-3’
Post-recombination transcripts		
Mouse		
Iμ-Cμ	5’-ACCTGGGAATGTATGGTTGTGGCTT-3’	5’-GAAATGGTGCTGGGCAGGAA-3’
Iμ-Cδ	5’-ACCTGGGAATGTATGGTTGTGGCTT-3’	5’-GCACTCTGAGAGGAGGAACA-3’
Iμ-Cγ1	5’-ACCTGGGAATGTATGGTTGTGGCTT-3’	5′-ATGGAGTTAGTTTGGGCAGCA-3′
Iμ-Cα	5’-ACCTGGGAATGTATGGTTGTGGCTT-3’	5′-TAATCGTGAATCAGGCAG-3′
Iμ-Cε	5’-ACCTGGGAATGTATGGTTGTGGCTT-3’	5′-ACAGGGCTTCAAGGGGTAGA-3′
Secreted and transmembrane forms of IgM and IgD		
Human		
V_HDJ_H-*Cμm*	5’-GACACGGCYGTRTATTACTGTGCG-3’	5’-AGAGGCTCAGGAGGAAGAGG-3’
V_HDJ_H-*Cμs*	5’-GACACGGCYGTRTATTACTGTGCG-3’	5’-CTGTGTCGGACATGACCAGG-3’
V_HDJ_H-*Cδm*	5’-GACACGGCYGTRTATTACTGTGCG-3’	5’-CCACAAACGTGGACAGGGT-3’
V_HDJ_H-*Cδs*	5’-GACACGGCYGTRTATTACTGTGCG-3’	5’-CATGGGGCCATGGTCTGTTACA-3’
Mouse		
V_HDJ_H-*Cμm*	5’-GCCTGACATCTGAGGACTCTGC-3’	5’-GCCTTCCTCCTCAGCATTCACCTC-3’
V_HDJ_H-*Cμs*	5’-GCCTGACATCTGAGGACTCTGC-3’	5’-CATGATCAGGGAGACATTGTACAG-3’
V_HDJ_H-*Cδm*	5’-GCCTGACATCTGAGGACTCTGC-3’	5’-ACACGAGTGTTGGATGGTGTTGAC-3’
V_HDJ_H-*Cδs*	5’-GCCTGACATCTGAGGACTCTGC-3’	5’-GGGCAGGACCATCAGGTTT-3’
S-S recombination		
Human		
Sμ-σδ		
First round	5’-TACCCTCCTCTTGGTGCAGA-3’	5’-CTGGCCAGCGGAAGATCTCCTTCTT-3’
Second round	5’-TGCTGCCACTTCTAGAGCAA-3’	5’-AGGGCTGTTATCCTTTGGGTG-3’
Sμ probe	5’-CCCCAGCCCTTGTTAATGGA-3’	5’-CCAGTGGGGCTTGGTATGTT-3’
σδ probe	5’-ACCAAAGCCTCTGGAGGGAA-3’	5’-AGGGCTGTTATCCTTTGGGTG-3’
Sμ-Sγ1		
First round	5’-TACCCTCCTCTTGGTGCAGA-3’	5’-AGTCAGCACAGTCCAGTGTCTCTAG-3’
Second round	5’-TGCTGCCACTTCTAGAGCAA-3’	5’-CATCGGTGCCACCTCAGGGACGCT-3’

Sμ probe	5'-CCCCAGCCCTTGTTAATGGA-3'	5'-CCAGTGGGGCTTGGTATGTT-3'
Sγ1 probe	5'-CACTGGGGCTAAGGGGAAAG-3'	5'-GCCCCACTCCAGCCTTTTAT-3'
Sμ-Sα1		
First round	5'-TACCCTCCTCTTGGTGCAGA-3'	5'-CTTTCGCTCCAGGTCACACT-3'
Second round	5'-TGCTGCCACTTCTAGAGCAA-3'	5'-TACTGGAGGAACCCAGCACA-3'
Sμ probe	5'-CCCCAGCCCTTGTTAATGGA-3'	5'-CCAGTGGGGCTTGGTATGTT-3'
Sα1 probe	5'-CTCTCTGTGCTGGGTTCCTC-3'	5'-TGTAGTGCTTCACGTGGCAT-3'
Mouse		
Sμ-σδ		
First round	5'-GGGCTTCTAAGCCAGTCCAC-3'	5'-CCAATTACTAAACAGCCCAGGT-3'
Second round	5'-CTCTGGCCCTGCTTATTGTTG-3'	5'-CAGCCCAGGTTTATCTTTTCA-3'
Sμ probe	5'-CTGGGAATGTATGGTTGTGGC-3'	5'-TGACCCAGACAACGGTACTC-3'
σδ probe	5'-CCCAGAACCTGAGAAGGAAG-3'	5'-CAGCCCAGGTTTATCTTTTCA-3'
Sμ-Sγ1		
First round	5'-GGGCTTCTAAGCCAGTCCAC-3'	5'-GGACAGGACAGGACCAAACC-3'
Second round	5'-CTCTGGCCCTGCTTATTGTTG-3'	5'-TAGAAGGCCGCTCTTTTGCA-3'
Sμ probe	5'-CTGGGAATGTATGGTTGTGGC-3'	5'-TGACCCAGACAACGGTACTC-3'
Sγ1 probe	5'-GTGCCGACTTCAATGTGCTT-3'	5'-CCCATGTCCCCGACTCTCTA-3'
Sμ-Sγ3		
First round	5'-GGGCTTCTAAGCCAGTCCAC-3'	5'-CTTTGACAAGGCATCCCAGTGT-3'
Second round	5'-CTCTGGCCCTGCTTATTGTTG-3'	5'-ACCAAGGGATAGACAGATGGGG-3'
Sμ probe	5'-CTGGGAATGTATGGTTGTGGC-3'	5'-TGACCCAGACAACGGTACTC-3'
Sγ3 probe	5'-AAGCACAGGTGCAAGAGACT-3'	5'-ACCAAGGGATAGACAGATGGGG-3'
Sμ-Sα		
First round	5'-GGGCTTCTAAGCCAGTCCAC-3'	5'-CATCCAATTCTTGGACGGCG-3'
Second round	5'-CTCTGGCCCTGCTTATTGTTG-3'	5'-CGGCGTTAGAGTCATGTTGC-3'
Sμ probe	5'-CTGGGAATGTATGGTTGTGGC-3'	5'-TGACCCAGACAACGGTACTC-3'
Sα probe	5'-ACCCAGTGATAATCGGCTGC-3'	5'-CGGCGTTAGAGTCATGTTGC -3'
Sμ-Sε		
First round	5'-GGGCTTCTAAGCCAGTCCAC-3'	5'-TCCACATGCCCAGGACATTC-3'
Second round	5'-CTCTGGCCCTGCTTATTGTTG-3'	5'-TTCTCCTGAGAGAGGGGCTC-3'
Sμ probe	5'-CTGGGAATGTATGGTTGTGGC-3'	5'-TGACCCAGACAACGGTACTC-3'
Sε probe	5'-GGTGGGGTTGAGCTGAATGA-3'	5'-ATTCCTGCTAGGCCCGATTG-3'
ChIP Assays		
Mouse Sμ	5'-ACCGCAAATGGTAAGCCAGA-3'	5'-TGTGAGTGACCCAGACAACG-3'
Mouse σδ	5'-ATGCCAACCCTGATTCAGCA-3'	5'-AGGCTAGGAGTCTGGGCTAC-3'
Mouse Sγ1	5'-AACCACAGAAGAGCAGGAGC-3'	5'-TACCCCGTACTCTCACCTGG-3'
Mouse Sγ3	5'-AGGGGACCTGGATAAGCCAT-3'	5'-CCCCACTATGGTTGCTTGGT-3'
Mouse Sα	5'-CTGGGCTGGACTCAGTTGAC-3'	5'-AGTCCAGTCATGCTAATTCACC-3'

Supplementary Table 2. Antibodies used in this study.

Antibody	Host	Type	Vendor	Cat. No. (mAb clone)	Assay	Working dilution
BV421-anti-mouse CD19	Rat	mAb	BD Biosciences	562701 (1D3)	FCM	1:1000
PE-anti-mouse IgM	Rat	mAb	BioLegend	406507 (RMM1)	FCM	1:1000
PerCP/Cyanine5.5-anti-mouse IgM	Rat	mAb	BioLegend	406512 (RMM1)	FCM	1:1000
APC-anti-mouse IgD	Rat	mAb	BioLegend	405713 (11-26c.2a)	FCM	1:1000
PE/Cyanine7-anti-mouse CD138	Rat	mAb	BioLegend	142513 (281-2)	FCM	1:1000
PE/Cyanine7-anti-human CD19	Mouse	mAb	BioLegend	302216 (HIB19)	FCM	1:100
APC/Fire™ 750-anti-human IgM	Mouse	mAb	BioLegend	314545 (MHM-88)	FCM	1:100
BV421-anti-human IgD	Mouse	mAb	BD Biosciences	562518 (IA6-2)	FCM	1:100
BV785™-anti-human IgD	Mouse	mAb	BioLegend	348241 (IA6-2)	FCM	1:100
PE-anti-human CD27	Mouse	mAb	BioLegend	356405 (M-T271)	FCM	1:200
BV650™-anti-human CD38	Mouse	mAb	BioLegend	356619 (HB-7)	FCM	1:100
APC-anti-human/mouse Zfp318	Rabbit	pAb	Aviva Systems Bio.	ARP32523_P050	FCM	1:200
FITC-anti-mouse IgD	Rat	mAb	BioLegend	405703 (11-26c.2a)	FCM	1:100
PE-anti-mouse Blimp-1	Rat	mAb	BioLegend	150005 (5E7)	FCM	1:100
Alexa Fluor® 647-anti-human IgD	Mouse	mAb	BioLegend	348227 (IA6-3)	FCM	1:50
Alexa Fluor® 488-anti-hu BLIMP1	Mouse	mAb	R&D SYSTEMS	646702 (IC36081G)	FCM	1:50
PE-anti-mouse IgD	Rat	mAb	BioLegend	405705 (11-26c.2a)	FCM	1:1000
PE-anti-mouse IgA	Rat	mAb	Invitrogen	12-4204-82 (mA-6E1)	FCM	1:1000
FITC-anti-mouse IgD	Rat	mAb	BioLegend	405704(11-26c.2a)	IF	1:200
FITC-anti-human IgD	Mouse	mAb	BioLegend	348205(IA6-2)	IF	1:100
PE-anti-mouse IgM	Rat	mAb	BioLegend	406507(RMM1)	IF	1:200
FITC-anti-mouse IgA	Rat	mAb	Invitrogen	12-4204-82 (mA-6E1)	IF	1:200
Anti-human/mouse AID	Rabbit	pAb	Cell Signaling	4949S (30F12)	IB	1:500
Anti-human/mouse Rad52	Rabbit	pAb	AbClonal	A3077	IB	1:500
Anti-human/mouse p-Rad52	Rabbit	mAb	Applied Biol. Mater.	Y408472 (Y104)	IB	1:1000
Anti-human/mouse Ku86	Rabbit	pAb	AbClonal	A5862	IB	1:1000
Anti-human/mouse Ku70	Rabbit	pAb	AbClonal	A0883	IB	1:1000
Anti-human/mouse β-Actin	Mouse	mAb	Sigma-Aldrich	A5441 (AC-15)	IB	1:1000
HRP-anti-rabbit IgG	Mouse	mAb	Santa Cruz Biot.	sc-2357-CM	IB	1:6000
HRP-anti-mouse IgG	Horse	pAb	Vectorlabs	PI-2000	IB	1:8000
Anti-mouse IgG	Goat	pAb	SouthernBiotech	1030-01	ELISA	1:1000
Anti-mouse IgA	Goat	pAb	SouthernBiotech	1040-01	ELISA	1:500
Anti-mouse IgM	Goat	pAb	SouthernBiotech	1020-01	ELISA	1:1000
Anti-mouse IgD	Goat	pAb	American Res. Pro.	21-1281	ELISA	1:5000
BIOT-anti-mouse IgG1	Goat	pAb	SouthernBiotech	1070-08	ELISA	1:5000
BIOT-anti-mouse IgA	Goat	pAb	SouthernBiotech	1040-08	ELISA	1:5000
BIOT-anti-mouse IgM	Goat	pAb	SouthernBiotech	1020-08	ELISA	1:5000
BIOT-anti-mouse IgD	Goat	pAb	SouthernBiotech	1120-08	ELISA	1:5000
Anti-human IgD	Goat	pAb	SouthernBiotech	2030-01	ELISA	1:1000
BIOT-anti-human IgD	Goat	pAb	SouthernBiotech	2030-08	ELISA	1:5000
Anti-human/mouse Rad52	Mouse	mAb	Santa Cruz Biot.	sc-365341 (F-7)	ChIP	1:100
Anti-human/mouse Ku70/Ku86	Mouse	mAb	Thermo Fisher Sci.	MA1-21818	ChIP	1:50
Isotype mouse IgG control	Mouse	pAb	Santa Cruz Biot.	sc-2025	ChIP	1:80

Abbreviations: mAb, monoclonal antibody; pAb, polyclonal antibody; ELISA, enzyme-linked immunosorbent assay; FCM, flow cytometry; IB, immunoblotting; IF, immunofluorescence; ChIP, chromatin immunoprecipitation.
