# Supplementary material for: Rad52 mediates class-switch DNA recombination to IgD
Source: Nat Commun. 2022 Feb 21;13:980. doi: 10.1038/s41467-022-28576-2 (PMC8861003; doi:10.1038/s41467-022-28576-2)
Supplement: Supplementary file 2 — Reporting Summary [file 41467_2022_28576_MOESM2_ESM.pdf]

nature portfolio

Corresponding author(s): Paolo Casali, M.D.

Last updated by author(s): December 20, 2021

Reporting Summary

Nature Portfolio wishes to improve the reproducibility of the work that we publish. This form provides structure for consistency and transparency in reporting. For further information on Nature Portfolio policies, see our Editorial Policies and the Editorial Policy Checklist.

Statistics

For all statistical analyses, confirm that the following items are present in the figure legend, table legend, main text, or Methods section.

n/a	Confirmed	
☐	☒	The exact sample size (*n*) for each experimental group/condition, given as a discrete number and unit of measurement
☐	☒	A statement on whether measurements were taken from distinct samples or whether the same sample was measured repeatedly
☐	☒	The statistical test(s) used AND whether they are one- or two-sided<br>*Only common tests should be described solely by name; describe more complex techniques in the Methods section.*
☒	☐	A description of all covariates tested
☒	☐	A description of any assumptions or corrections, such as tests of normality and adjustment for multiple comparisons
☐	☒	A full description of the statistical parameters including central tendency (e.g. means) or other basic estimates (e.g. regression coefficient) AND variation (e.g. standard deviation) or associated estimates of uncertainty (e.g. confidence intervals)
☒	☐	For null hypothesis testing, the test statistic (e.g. *F*, *t*, *r*) with confidence intervals, effect sizes, degrees of freedom and *P* value noted<br>*Give P values as exact values whenever suitable.*
☒	☐	For Bayesian analysis, information on the choice of priors and Markov chain Monte Carlo settings
☒	☐	For hierarchical and complex designs, identification of the appropriate level for tests and full reporting of outcomes
☒	☐	Estimates of effect sizes (e.g. Cohen's *d*, Pearson's *r*), indicating how they were calculated

Our web collection on statistics for biologists contains articles on many of the points above.

Software and code

Policy information about availability of computer code

Data collection	- Quant Studio Real-Time PCR Software v1.5.1<br>- BD FACSDiva Software version 8.0.1<br>- FlowJo Software version 10.6.2<br>- Illumina HiSeq 3000 version HCS 3.4.0<br>- Illumina MiSeq version MCS v3.1
Data analysis	- FlowJo Software version 10.6.2<br>-ImageJ v1.50i<br>-MacVector v15.03

For manuscripts utilizing custom algorithms or software that are central to the research but not yet described in published literature, software must be made available to editors and reviewers. We strongly encourage code deposition in a community repository (e.g. GitHub). See the Nature Portfolio guidelines for submitting code & software for further information.

Data

Policy information about availability of data

All manuscripts must include a data availability statement. This statement should provide the following information, where applicable:

- Accession codes, unique identifiers, or web links for publicly available datasets
- A description of any restrictions on data availability
- For clinical datasets or third party data, please ensure that the statement adheres to our policy

Provide your data availability statement here.

Field-specific reporting

Please select the one below that is the best fit for your research. If you are not sure, read the appropriate sections before making your selection.

☒ Life sciences ☐ Behavioural & social sciences ☐ Ecological, evolutionary & environmental sciences

For a reference copy of the document with all sections, see nature.com/documents/nr-reporting-summary-flat.pdf

Life sciences study design

All studies must disclose on these points even when the disclosure is negative.

Sample size	No sample-size calculations were performed. Sample sizes were chosen to provide enough replicates for statistical analysis and were determined by availability of biological samples and/or based on experience with our biological models.
Data exclusions	No data exclusion.
Replication	All figure legends of experimental data contain clear descriptions of the biological and technical replicates.
Randomization	The samples analyzed in this study were randomly chosen.
Blinding	Blinding was not performed.

Reporting for specific materials, systems and methods

We require information from authors about some types of materials, experimental systems and methods used in many studies. Here, indicate whether each material, system or method listed is relevant to your study. If you are not sure if a list item applies to your research, read the appropriate section before selecting a response.

Materials & experimental systems

n/a	Involved in the study	
☐	☒	Antibodies
☒	☐	Eukaryotic cell lines
☒	☐	Palaeontology and archaeology
☐	☒	Animals and other organisms
☐	☒	Human research participants
☒	☐	Clinical data
☒	☐	Dual use research of concern

Methods

n/a	Involved in the study	
☒	☐	ChIP-seq
☐	☒	Flow cytometry
☒	☐	MRI-based neuroimaging

Antibodies

Antibodies used	Antibodies for flow cytometry: BV421-anti-CD19 mAb (clone 1D3, BD), PE-anti-IgM mAb (clone RMM1, 406507, BioLegend), FITC-anti-mouse IgD mAb (clone 11-26c.2a, 405704, BioLegend), anti-Zfp318 Ab (ARP32523_P050, Aviva Systems Biology; labeled with FITC using iLink™ Antibody Labeling Kits, ABP Biosciences), FITC-anti-IgA mAb (C10-3, BD Biosciences), PE-anti-Blimp1 mAb (5E7, BioLegend), PE-Cy7-anti-mouse CD138 mAb (Clone 281-2, 142513, BioLegend), AlexaFluor488-anti-human BLIMP1 Ab (IC36081G, R&D Systems), BV510-anti-human CD138 Ab (356517, BioLegend), BV421-mouse-anti-human IgD mAb (Clone IA6-2, 562518, BD Horizon), BV785™-anti-human IgD mAb (clone IA6-2, 348241, BioLegend), PE/Cyanine7 anti-human CD19 mAb (clone HIB19, 302216, BioLegend), APC/Fire™ 750-anti-human IgM mAb (clone MHM-88, 314545, BioLegend), PE-anti-human CD27 mAb (clone M-T271, 356405, BioLegend), or BV650™-anti-human CD38 mAb (clone HB-7, 356619, BioLegend), all at concentration of 0.5 μg per million cells in 100 μl volume.<br><br>Antibody for B cell positive selection: biotin-anti-human IgD mAb (clone IA6-2; 348212, Biolegend), biotin-anti-human IgD mAb (clone IA-6-2, 348212, BioLegend), all at concentration of 0.25 μg per million cells in 100 μl.

	Antibodies for immunoblotting: anti-AID antibody (H-80, Santa Cruz), anti-Ku70 antibody (A0883, Abclonal), anti-Ku86 antibody (A5862, Abclonal), anti-Rad52 antibody (H-300, Santa Cruz Biotechnology), anti-phospho-Rad52 antibody (Y408472, Applied Biological Materials Inc.), anti-β-Actin mAb (2F1-1, BioLegend), HRP anti-rabbit IgG mouse mAb (clone 6B9G9, 410406 BioLegend), HRP Goat anti-mouse IgG (minimal x-reactivity) antibody (405306, BioLegend), all at concentration of 0.5 μg per million cells in 100 μl.<br><br>Antibodies for ELISA and dot-blotting: Biotin-anti-mouse IgD mAb (Clone 11-26c.2a, 405733, BioLegend), anti-human IgD mAb (clone W18340A, 324502, BioLegend), Biotin-anti-human IgD mAb (Clone IA6-2, 348212, BioLegend).<br><br>Antibodies for ChIP assays: Agarose conjugated anti-Rad52 mAb (clone F-7; sc-365341 AC, Santa Cruz Biotechnology), anti-Ku70/86 mAb (MA1-21818, Thermo Fisher Scientific), all at concentration of 5 μg ml−1.<br><br>Antibodies for fluorescence microscopy: FITC–anti-IgD mAb (clone 11-26c.2a; 405713, BioLegend), PE-anti-mouse IgM mAb (clone II/41, 12-5790-82, Invitrogen) and FITC–anti-mouse IgA mAb (clone mA-6E1, 11-4204, Invitrogen), FITC–anti-IgD mAb (405704, BioLegend), or PE goat-anti-mouse-IgM mAb (406507, BioLegend), all at concentration of 1 μg ml−1.
Validation	All antibodies are validated according to manufacturer information.

Animals and other organisms

Policy information about studies involving animals; ARRIVE guidelines recommended for reporting animal research

Laboratory animals	C57BL/6, MRL/Fas(lpr/lpr), Rad52(-/-) and Aicda(-/-) mice were used.
Wild animals	No wild animal was involved in this study.
Field-collected samples	No field-collected sample was used in this study.
Ethics oversight	All animal experiments were conducted in accordance with the guidelines and approved by the Institutional Animal Care and Use Committee (IACUC) of the University of Texas Health Science Center at San Antonio.

Note that full information on the approval of the study protocol must also be provided in the manuscript.

Human research participants

Policy information about studies involving human research participants

Population characteristics	De-identified formalin fixed paraffin embedded IgD and IgA myeloma tissue samples were obtained from the University of Arkansas for Medical Science. These myeloma tissue samples were identified based on SPEP/IF and further confirmed by Sm-sd or Sm-Sa DNA recombination as indicated by nested PCR and Southern-blotting. Peripheral blood B cells of healthy subjects were isolated from buffy coats obtained from the South Texas Blood & Tissue Center (STBTC). SLE blood samples were collected from patients who meet at least 4 of the 11 ACR classification criteria for this disease, and whose disease is active, as defined by an SLE Disease Activity Index (SLEDAI) of 3 or greater.
Recruitment	Peripheral blood B cells of healthy subjects were isolated from buffy coats obtained from the South Texas Blood & Tissue Center (STBTC). The healthy subjects were enrolled and consented by STBTC. The investigators have no interaction with these subjects and have not been provided with any personal information of these subjects. Blood from SLE patients was collected at the Medical Arts & Research Center (MARC) of UTHSCSA. The authors do not have personal information of these patients.
Ethics oversight	For the use of DNA procured from formalin fixed paraffin embedded tissues obtained from the University of Arkansas for Medical Science, the study was reviewed by the University of Arkansas for Medical Sciences Institutional Review Board (IRB) which determined that this project is not human subject research as defined in 45 CFR 46.102. Human B cells were purified from PBMCs of healthy subject buffy coats obtained from South Texas Blood and Tissue Center, San Antonio, Texas, under the Healthy Volunteer Blood Donor Program and lupus patients B cells were purified PBMCs obtained under the UT Health San Antonio IRB HSC 20140234H.

Note that full information on the approval of the study protocol must also be provided in the manuscript.

Flow Cytometry

Plots

Confirm that:

- ☒ The axis labels state the marker and fluorochrome used (e.g. CD4-FITC).
- ☒ The axis scales are clearly visible. Include numbers along axes only for bottom left plot of group (a 'group' is an analysis of identical markers).
- ☒ All plots are contour plots with outliers or pseudocolor plots.
- ☒ A numerical value for number of cells or percentage (with statistics) is provided.

Methodology

Sample preparation	For surface staining, mouse mononuclear cells were reacted with BV421-anti-CD19 mAb (clone 1D3, BD), PE-anti-IgM mAb (clone RMM1, 406507, BioLegend), PerCP/Cyanine5.5 -anti-IgM mAb (clone RMM1, 406512, BioLegend), FITC anti-mouse IgD mAb (clone 11-26c.2a, 405704, BioLegend), PE/Cyanine7-anti-mouse CD138 mAb (clone 281-2, 142513, BioLegend), or 7-AAD. Human mononuclear cells were reacted with PE/Cyanine7 anti-human CD19 mAb (clone HIB19, 302216, BioLegend), APC/Fire™ 750-anti-human IgM mAb (clone MHM-88, 314545, BioLegend), BV421-anti-human IgD mAb (clone IA6-2, 562518, BD Horizon™), BV785™-anti-human IgD mAb (clone IA6-2, 348241, BioLegend), PE-anti-human CD27 mAb (clone M-T271, 356405, BioLegend), or BV650™-anti-human CD38 mAb (clone HB-7, 356619, BioLegend).<br>For intracellular staining, mouse and human cells, pre-stained for select surface markers and treated with Fixable Viability Dye eFluor™ 780(Fisher Scientific), were incubated with the BD Cytofix/Cytoperm buffer at 4°C for 45 min. After washing twice with the BD Perm/Wash buffer, cells were resuspended in HBSS with 1% BSA and stored overnight at 4°C. Mouse cells were then stained with FITC-anti-Zfp318 Ab (ARP32523_P050, Aviva Systems Biology; labeled with FITC using iLink™ Antibody Labeling Kits, ABP Biosciences), FITC-anti-mouse IgD mAb (clone 11-26c.2a, 405703, BioLegend) or PE anti-mouse Blimp-1 mAb (clone 5E7, 150005, BioLegend). Human cells were then stained with FITC-anti-Zfp318 Ab, Alexa Fluor® 647-anti-human IgD mAb (clone IA6-3, 348227, BioLegend) or Alexa Fluor® 488-anti-human BLIMP1 mAb (clone 646702, IC36081G, R&D Systems).
Instrument	Flow cytometry analyses were performed using a LSR-II or Celesta flow cytometer (BD Biosciences).
Software	Flow cytometry data were analyzed using FlowJo software (TreeStar).
Cell population abundance	No FACS sorting were done. Human and mouse B cells were purified using the EasySep™ Human Naïve B Cell Enrichment Kit or the EasySep™ Mouse B Cell Isolation Kit (STEMCELL Technologies), respectively. The purity of these B cells was at least 98%, as analyzed for CD19+ cells by flow cytometry.
Gating strategy	FACS analyses were performed on single cell suspensions. In all flow cytometry experiments, cells were appropriately gated by forward and side-scattering to exclude dead cells and debris.

☒ Tick this box to confirm that a figure exemplifying the gating strategy is provided in the Supplementary Information.
